# Bis(imino)pyridine iron complexes for catalytic carbene transfer reactions†
†Electronic supplementary information (ESI) available: Experimental details and copies of NMR spectra. See DOI: 10.1039/c9sc02189b


**DOI:** 10.1039/c9sc02189b

**Published:** 2019-07-02

**Authors:** Ban Wang, Isaac G. Howard, Jackson W. Pope, Eric D. Conte, Yongming Deng

**Affiliations:** a Chemistry Department , Western Kentucky University , 1906 College Heights Boulevard , Bowling Green , Kentucky 42101 , USA . Email: yongming.deng@wku.edu

## Abstract

The bis(imino)pyridine iron complex, for the first time, is developed as an effective metal carbene catalyst for carbene transfer reactions of donor–acceptor diazo compounds. Its broad catalytic capability is demonstrated by a range of metal carbene reactions, from cyclopropanation, cyclopropenation, epoxidation, and Doyle–Kirmse reaction to O–H insertion, N–H insertion, and C–H insertion reactions. The asymmetric cyclopropanation of styrene and methyl phenyldiazoacetate was successfully achieved by the new chiral bis(imino)pyridine iron catalyst, which delivers a new gateway for the development of chiral iron catalysis for metal carbene reactions.

## Abstract 2

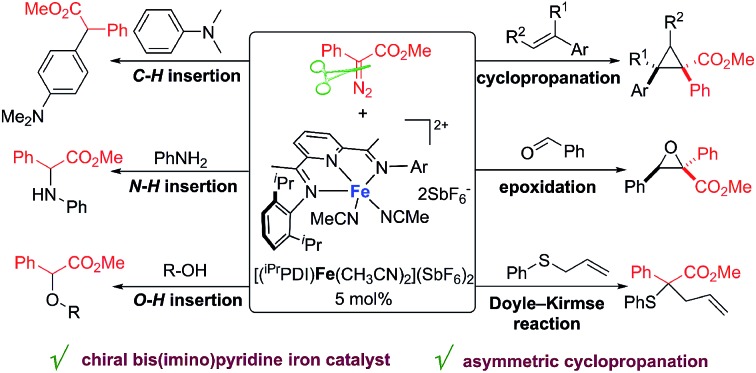
The bis(imino)pyridine iron complex, for the first time, is developed as an effective metal carbene catalyst for carbene transfer reactions of donor–acceptor diazo compounds.

## Introduction

Transition-metal-catalyzed carbene transfer reactions occurring through metal carbene intermediates encompass a vast array of reactants and catalysts to achieve novel and selective strategies for organic synthesis.^1^ The reactive carbenoid intermediates can be catalytically generated from diazo compounds by metal-catalyzed dinitrogen extrusion,^2^ and their reactions extend from addition and insertion to cycloaddition and ylide formation.^3^ Dirhodium complexes have been established as the most successful catalysts for carbene transfer reactions of diazo compounds;^4^ great achievements have also been accomplished recently by copper and other precious metal catalysts (*e.g.* ruthenium, palladium, gold).^5^ Iron, the second most abundant metal, with its particular biological relevance, is emerging as an important metal for catalytic metal carbene reactions.^6^ However, iron catalysis is comparatively underdeveloped, with the enduring dominance of precious metal catalysis in metal carbene chemistry.

**Scheme 1:**
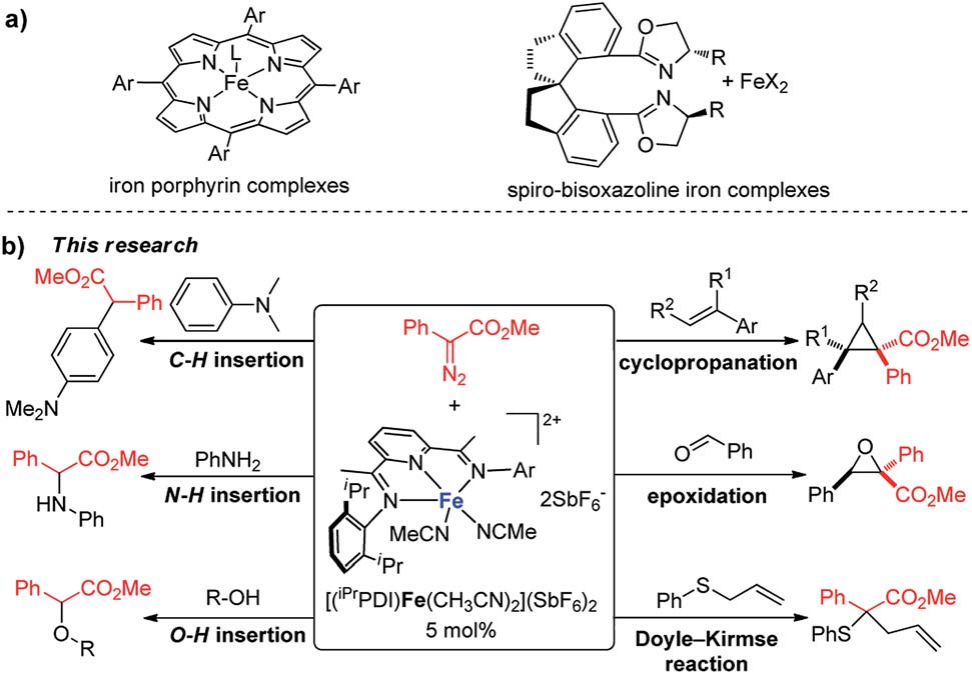
(a) Selected iron catalysis for metal carbene reactions. (b) This research: bis(imino)pyridine iron catalyzed metal carbene reactions.

Since the launch of iron porphyrin-catalyzed cyclopropanation by Woo,^7^ various carbene transfer processes of diazo compounds, including cyclopropanation, heteroatom–hydrogen bond insertions, and intramolecular C–H inversion, have been achieved by porphyrin and related macrocyclic iron complexes; however, these generally occur with active α-hydrogen-diazocarbonyl compounds, diazoalkanes, or the corresponding precursors.^8^ The spiro-bisoxazoline iron complexes developed by Zhou's group have exhibited high catalytic activities and selectivities for heteroatom–hydrogen bond insertions and intramolecular cyclopropanation reactions of α-diazoesters.^9^ Despite these achievements, iron has not been developed as a catalyst to the same extent as other late transition metals, particularly for usage in metal carbene reactions. The advancement of iron catalysis for general carbene transfer reactions with broad substrate schemes, especially asymmetric processes and under mild reaction conditions, remains a wide-open field for discovery and innovation. We report here, for the first time, bis(imino)pyridine iron complexes serving as effective catalysts for a range of metal carbene reactions under mild reaction conditions (at room temperature or 40 °C), including cyclopropanation/cyclopropenation, epoxidation, Doyle–Kirmse reaction, O–H insertion, N–H insertion, and C–H insertion (Scheme 1). To the best of our knowledge, this bis(imino)pyridine iron catalyst represents the most broad-ranging catalytic activity towards metal carbene reactions of diazo compounds over the previously reported iron catalysis system.^6^ The bis(imino)pyridine iron-catalyzed cyclopropanation proceeds on a wide range of aryldiazoacetates, vinyldiazoacetates, styrenes and phenylacetylene. Notably, a new chiral bis(imino)pyridine ligand derivatized from l-valine methyl ester has been synthesized, and the corresponding enantiopure, *C*_1_-symmetric iron catalyst enabled the asymmetric cyclopropanation of styrene and phenyl diazoacetate.

**Fig. 1:**
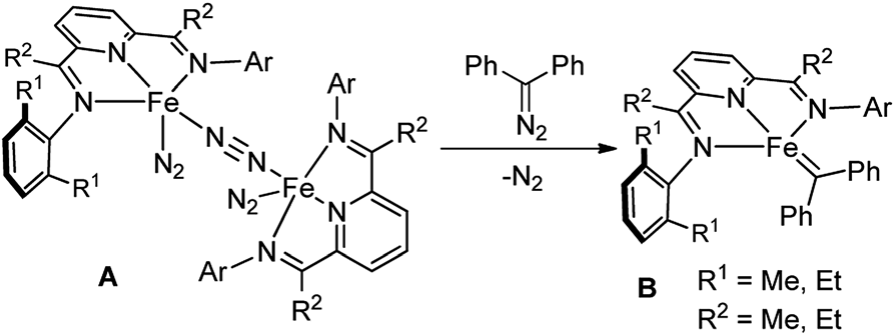
Formation of a bis(imino)pyridine iron carbene from bis(arylimino)pyridine iron dinitrogen complexes and diphenyldiazomethane.^11^

In the past decade, bis(imino)pyridine chelated iron complexes have emerged as an effective class of catalysts for ethylene polymerization, olefin hydrogenation, hydrosilation, and [2π + 2π]-cycloaddition reactions.^10^ Owing to its ease of preparation, the bis(imino)pyridine ligand is easily modifiable, allowing versatility in ligand design, synthesis, and screening.10a,b However, catalytic metal carbene reactions by bis(imino)pyridine iron complexes have not been achieved. Recently, Chirik reported the formation of a bis(imino)pyridine iron carbene complex **B** from a stoichiometric amount of bis(imino)pyridine iron dinitrogen complex **A** and diphenyldiazomethane by dinitrogen extrusion (Fig. 1).^11^ However, the attempts towards metal carbene reactions, such as cyclopropanation and C–H insertion, were unsuccessful with this bis(imino)pyridine iron carbene complex.^11^,^12^

We hypothesized that one reason for the lack of reactivity for bis(imino)pyridine iron carbene complex **B** in the carbene transfer process is due to the charge delocalization induced by the diphenyl group. To address this issue, we predicted that augmenting the electrophilicity of the disubstituted diazo compound would increase the reactivity of the corresponding iron carbene; thus, it could more readily engage in carbene transfer reactions.2b,c It has been documented that the donor–acceptor metal carbene, which can be produced from donor–acceptor diazo compound by metal-catalyzed dinitrogen extrusion, exhibited higher reactivity than the one from diphenyldiazomethane due to its stronger electrophilicity.1c,3c,4a Herein, a donor–acceptor diazo compound, aryldiazoacetate, was selected as the carbene precursor to investigate the bis(imino)pyridine iron-catalyzed metal carbene reactions. Additionally, recent computational studies of bis(imino)pyridine iron complexes for C–H functionalization of donor–acceptor diazo compound also suggest feasibility.^13^

The catalytic cycle for the conversion of a diazo compound to a metal-stabilized carbene intermediate is initiated from the metal-catalyzed dinitrogen extrusion of nucleophilic diazo compound. We predicted that compared to the formal iron(0) complex **A**, the more electrophilic bis(imino)pyridine iron(ii) complexes would exhibit higher reactivity towards the nucleophilic diazo compound and facilitate the subsequent metal carbene transfer. Therefore, we aimed to electronically and sterically tune the bis(imino)pyridine iron(ii) complexes to achieve the carbene transfer reactions of the donor–acceptor diazo compound under mild reaction conditions.

## Results and discussion

As a starting point, we focused on evaluating a series of bis(imino)pyridine iron(ii) catalysts for the cyclopropanation reaction of styrene **2a** with methyl phenyldiazoacetate **1a** (Table 1). As proposed, in the presence of 5 mol% of bis(arylimino)pyridine iron(ii) dichloride complexes (entries 1 and 2), the reaction of **2a** and phenyldiazoacetate **1a** afforded the cyclopropanation product **3a**, however, in low yields with predominately recovered starting material. To improve the catalytic activity of the iron complexes, examination of the noncoordinating counterions was performed. The employment of more electrophilic iron complexes with hexafluoroantimonate (SbF_6_^–^) as counterions (entries 3 and 4) led to a marked increase in yield. The combination of (^iPr^PDI)FeCl_2_ and NaBAr^F^_4_ also delivered **3a** with enhanced yield (48%, Table S1†). [(^iPr^PDI)Fe(CH_3_CN)_2_](SbF_6_)_2_ bearing bulky 2,6-diisopropylphenyl substituents was identified as the best catalyst,^14^ which catalyzed the cyclopropanation under room temperature, generating **3a** in 86% yield with excellent diastereoselectivity (dr > 20 : 1). A lower yield of **3a** (entry 5, 68%) was obtained when the reaction was performed with **1a** : **2a** = 1 : 1 in the presence of [(^iPr^PDI)Fe(CH_3_CN)_2_](SbF_6_)_2_. To reveal the imino-substituents' effect on the catalyst, iron complexes containing *N*-alkyl substituents were examined. However, they resulted in low catalytic activity with recovery of starting material (entries 6 and 7), which could be due to electronic and/or steric constraints from the imino-alkyl groups. Additionally, neither iron complexes of pyridine bis(oxazoline) ligands nor oxazoline iminopyridine iron complexes were effective catalysts for this transformation (entries 8 to 10). These results demonstrate the indispensability of the imino-aryl substituent in the ligand frame to conduct active iron catalysis in metal carbene reactions of donor–acceptor diazo compound.

**Table 1:** Screening of iron catalysts for cyclopropanation^*a*^

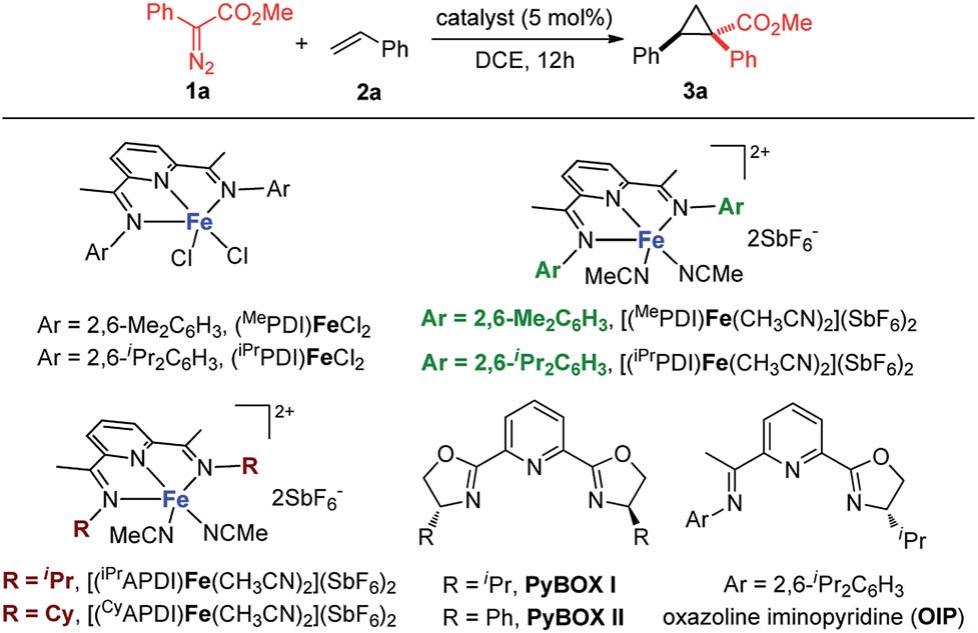			
Entry	Catalyst	T (°C)	Yield^*b*^
1	(^Me^PDI)FeCl_2_	50	14
2	(^iPr^PDI)FeCl_2_	50	18
3	[(^Me^PDI)Fe(CH_3_CN)_2_](SbF_6_)_2_	rt	65
**4**	**[(** ^**iPr**^ **PDI)Fe(CH**_**3**_ **CN)**_**2**_ **](SbF**_**6**_ **)**_**2**_	**rt**	**86**
5^*c*^	[(^iPr^PDI)Fe(CH_3_CN)_2_](SbF_6_)_2_	rt	68
6	[(^iPr^APDI)Fe(CH_3_CN)_2_](SbF_6_)_2_	50	8
7	[(^Cy^APDI)Fe(CH_3_CN)_2_](SbF_6_)_2_	50	<5
8	FeCl_2_/PyBOX(i)/AgSbF_6_	50	<5
9	FeCl_2_/PyBOX(ii)/AgSbF_6_	50	<5
10	FeCl_2_/OIP/AgSbF_6_	50	9

^*a*^Reaction condition unless otherwise noted: **1a** (0.20 mmol, 1.0 equiv.) in dry DCE (1.0 ml) was added to a 1.0 mL DCE solution of **2a** (1.0 mmol, 5.0 equiv.) and catalyst (0.01 mmol) under N_2_ within 1 hour.

^*b*^Yield of isolated product **3a** based on the limiting reagent **1a**.

^*c*^The reaction was performed with **1a** : **2a** = 1 : 1 (1,2-dichloroethane = DCE).

Under the optimized condition, we investigated the scope of this bis(arylimino)pyridine iron-catalyzed cyclopropanation across a range of aryldiazoacetates and styrene derivatives (Table 2). As indicated by entries 1 to 5, aryldiazoacetates with electron-rich, halogen *para*-substituents and 2-naphthyl group all reacted smoothly with styrene, generating the corresponding cyclopropanes in good yields (81–88%, **3b–3f**) with excellent diastereoselectivities (dr > 20 : 1). However, no reaction occurred with the electron-deficient system, even at 40 °C (**1g**, entry 6). Reactions of aryldiazoacetates **1h** and **1j** bearing *ortho*-substituents on the aromatic ring resulted in lower yields (entries 7 and 8). We rationalize that such lower reactivity can be attributed to a higher kinetic barrier for the generation of corresponding iron carbene intermediate, which is caused by the increased steric hindrance between the *ortho*-substituent and the bulky bis(imino)pyridine ligand frame. The cyclopropanes **3j–3l** derived from styrene derivatives **2b–2d** were obtained in yields ranging from 88 to 91%, whereas moderate yield (67%, entry 12) was obtained with 4-(trifluoromethyl)styrene **2e**. Disubstituted styrenes, including α-phenylstyrene **2f** and *trans*-β-methylstyrene **2g**, were also ideal reagents for this iron-catalyzed cyclopropanation, producing products **3n** and **3o** in good yields.

**Table 2:** Scope of bis(imino)pyridine iron-catalyzed cyclopropanation^*a*^

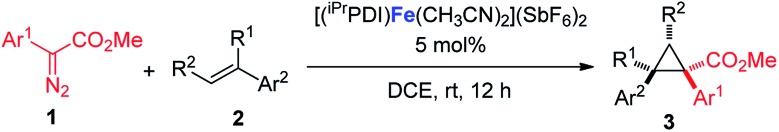			
Entry	**1**	**2**	Yield^*b*^
1	**1b**, 4-MeC_6_H_4_	**2a**, Ph, H, H	**3b**, 81
2	**1c**, 4-MeOC_6_H_4_	**2a**, Ph, H, H	**3c**, 83
3	**1d**, 4-ClC_6_H_4_	**2a**, Ph, H, H	**3d**, 88
4	**1e**, 4-BrC_6_H_4_	**2a**, Ph, H, H	**3e**, 83
5	**1f**, 2-naphthyl	**2a**, Ph, H, H	**3f**, 81
6^*c*^	**1g**, 4-NO_2_C_6_H_4_	**2a**, Ph, H, H	**3g**, <5
7	**1h**, 2-MeOC_6_H_4_	**2a**, Ph, H, H	**3h**, 52
8	**1i**, 2-ClCH_6_H_4_	**2a**, Ph, H, H	**3i**, 58
9	**1a**, Ph	**2b**, 4-MeC_6_H_4_, H, H	**3j**, 91
10	**1a**, Ph	**2c**, 4-MeOC_6_H_4_, H, H	**3k**, 88
11	**1a**, Ph	**2d**, 4-ClC_6_H_4_, H, H	**3l**, 90
12	**1a**, Ph	**2e**, 4-CF_3_C_6_H_4_, H, H	**3m**, 67
13^*c*^	**1a**, Ph	**2f**, Ph, Ph, H	**3n**, 73
14^*c*^	**1a**, Ph	**2g**, Ph, H, CH_3_	**3o**, 70

^*a*^For experimental details, see ESI.

^*b*^Isolated yield.

^*c*^Reactions were performed at 40 °C.

In addition to the styrene derivatives, the reaction of 1,3-cyclohexadiene and **1a** was also effectively catalyzed by [(^iPr^PDI)Fe(CH_3_CN)_2_](SbF_6_)_2_, affording the cyclopropane product **3p** in 80% yield with dr > 20 : 1 (eqn (1)). Furthermore, as shown in eqn (2), [(^iPr^PDI)Fe(CH_3_CN)_2_](SbF_6_)_2_ catalyzed the cyclopropanation of cyclohexene, and **1a** was also successfully achieved, affording the desired product **3q** in 72% yield. To further probe the diazo substrate generality, vinyl-diazoacetate **1j** was subjected to bis(imino)pyridine iron-catalyzed cyclopropanation with styrene (eqn (3)). Gratifyingly, the cyclopropane product **3r** was obtained in 84% yield, which demonstrates the catalytic capability of bis(imino)pyridine iron for a broader scope of donor–acceptor diazo compounds. Remarkably, [(^iPr^PDI)Fe(CH_3_CN)_2_](SbF_6_)_2_ was also capable of catalyzing the cyclopropenation of **1a** and phenylacetylene, furnishing the product **3s** in 61% yield at 40 °C (eqn (4)), which has not been achieved by other reported iron catalysts.1
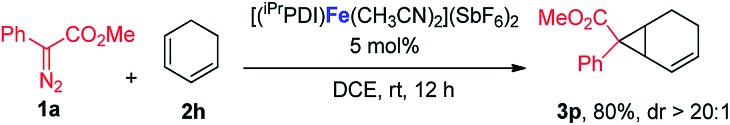

2
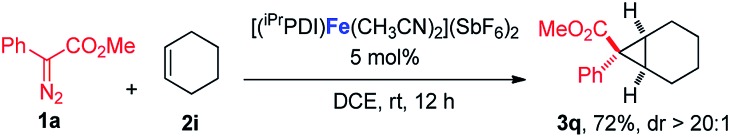

3
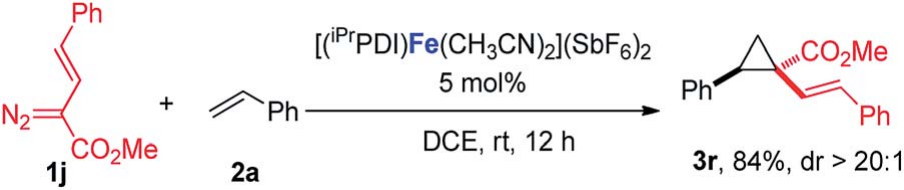

4
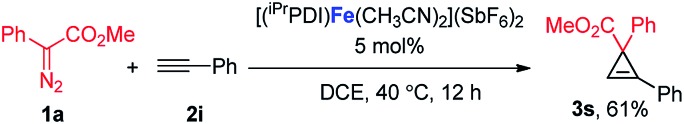



**Scheme 2:**
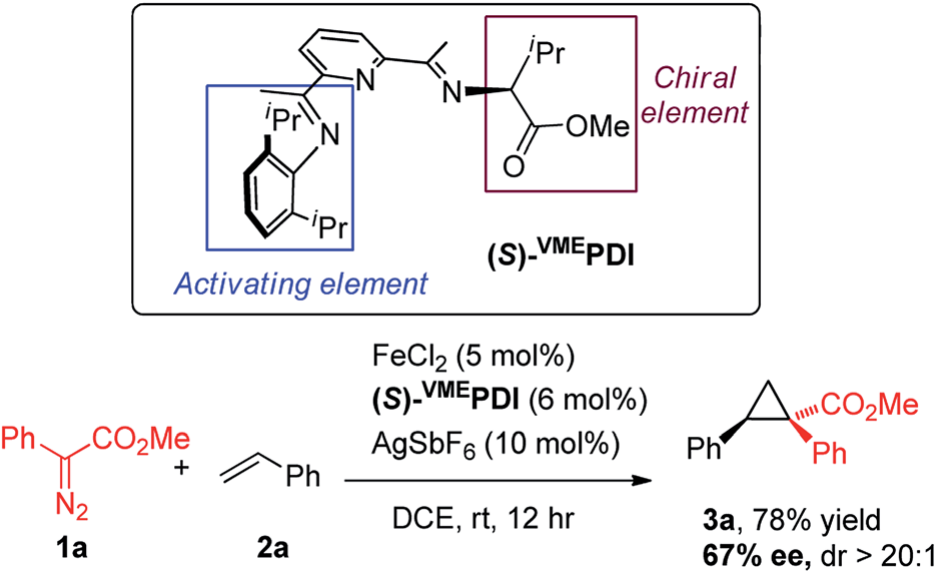
Chiral bis(imino)pyridine iron-catalyzed cyclopropanation.

With the accomplishment of achiral bis(arylimino)pyridine iron-catalyzed cyclopropanation, we have sought to modify the ligand architecture to generate a chiral iron catalyst for asymmetric cyclopropanation. Our catalyst screening (Table 1) indicated that the *N*-aryl substituent in bis(imino)pyridine ligand is indispensable for the effective catalytic activity of iron complexes. Guided by these experimental results and Bianchini's original design of chiral bis(imino)pyridine ligand,^15^ we synthesized an enantiopure, *C*_1_-symmetric chiral bis(imino)pyridine ligand [(*S*)-^VME^PDI] (Scheme 2), in which one imine is “anchored” by a 2,6-diisopropylphenyl group (activating element) and the other is prepared from l-valine methyl ester (chiral element). To our delight, the asymmetric cyclopropanation reaction of **1a** and styrene was successfully achieved by *in situ* prepared chiral iron catalyst from (*S*)-^VME^PDI, FeCl_2_, and AgSbF_6_ at room temperature. The cyclopropane product **1a** was isolated in 78% yield with 67% enantiomeric excess.^16^ Although with moderate enantioselectivity, the success of this asymmetric cyclopropanation reaction provides a strong basis for the development of a new chiral bis(imino)pyridine iron catalyst for metal carbene reactions.

**Scheme 3:**
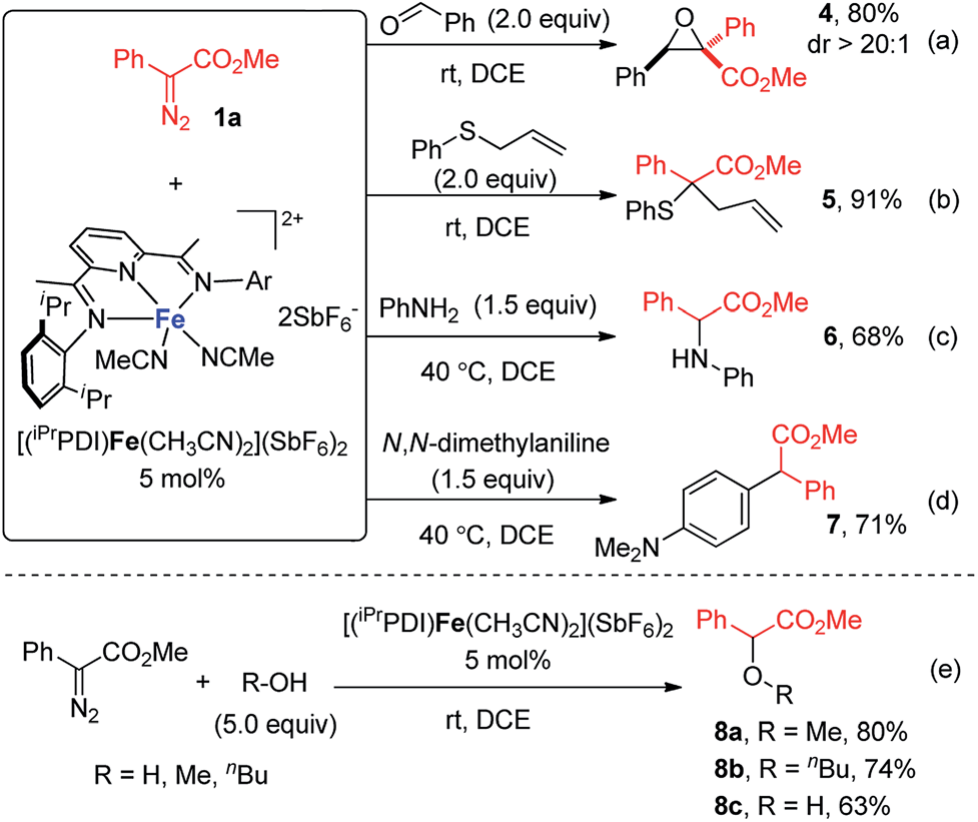
Bis(arylimino)pyridine iron-catalyzed (a) epoxidation; (b) Doyle–Kirmse reaction; (c) N–H insertion; (d) C–H insertion; and (e) O–H insertion.

Encouraged by the success of bis(arylimino)pyridine iron(ii)-catalyzed cyclopropanation, we then sought to examine the generality of this iron catalyst for metal carbene reactions. As depicted in Scheme 3, a range of metal carbene reactions of phenyldiazoacetate **1a**, including epoxidation, Doyle–Kirmse reaction, N–H insertion, C–H insertion, and O–H insertion, were all successfully catalyzed by [(^iPr^PDI)Fe(CH_3_CN)_2_](SbF_6_)_2_. The bis(arylimino)pyridine iron-catalyzed reaction of **1a** and benzaldehyde yielded the epoxide product **4** in 80% yield with excellent diastereoselectivity at room temperature (Scheme 3a). Under the same condition (Scheme 3b), allyl phenyl sulfide reacted with **1a** smoothly to form the Doyle–Kirmse product **5** in 91% yield. [(^iPr^PDI)Fe(CH_3_CN)_2_](SbF_6_)_2_ was also able to catalyze the N–H insertion of aniline and C–H insertion of *N*,*N*-dimethylaniline, although higher reaction temperatures were required (Scheme 3c and d). Furthermore, in the presence of 5 mol% [(^iPr^PDI)Fe(CH_3_CN)_2_](SbF_6_)_2_, O–H insertion reactions of **1a** with methanol, *n*-butanol, and water were achieved, furnishing the corresponding products **8a–8c** in good to moderate yields (Scheme 3e).

**Scheme 4:**
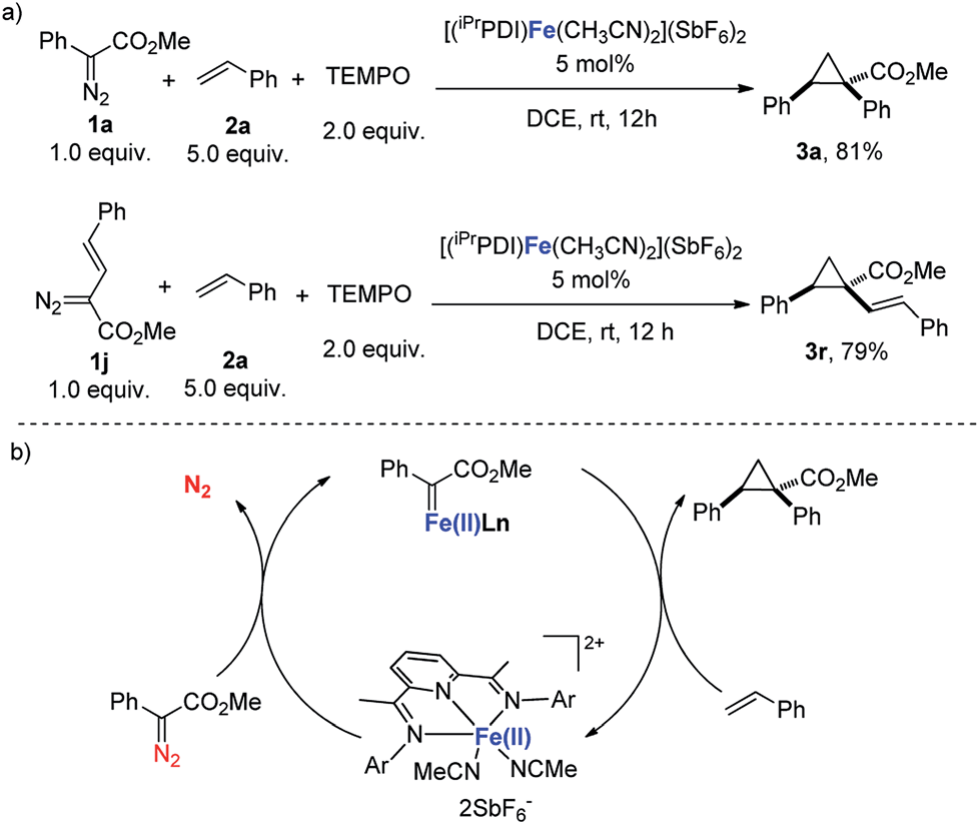
(a) Mechanism study. (b) Proposed mechanism of bis(arylimino)pyridine iron(ii)-catalyzed cyclopropanation.

As documented, bis(imino)pyridines have been recognized as radical-based, redox non-innocent ligands that can directly participate in the electronic structure of metal complexes.10d,f,^17^ Chirik's study demonstrated that a carbene radical is engaged in bis(imino)pyridine iron carbene complex **A**, which is obtained from a formal iron(0) complex (Scheme 2).^11^ Therefore, considering the redox activity of the bis(imino)pyridine ligand, radical tapping experiments were conducted to address whether a radical carbene involved in this bis(arylimino)pyridine iron(ii) catalyzes carbene transfer reactions.^18^ As shown in Scheme 4a, the addition of the radical scavenger TEMPO (2,2,6,6-tetramethylpiperidine *N*-oxide) did not harm the [(^iPr^PDI)Fe(CH_3_CN)_2_](SbF_6_)_2_-catalyzed cyclopropanation reactions of **1a** or vinyl-diazoacetate **1j**, and the corresponding products were isolated with similar yields to those from the reactions in the absence of TEMPO. These results reveal the unlikely involvement of the carbene radical intermediate in [(^iPr^PDI)Fe(CH_3_CN)_2_](SbF_6_)_2_-catalyzed cyclopropanation reactions. Moreover, the achievement of C–H insertion reaction of **1a** with *N*,*N*-dimethylaniline (Scheme 3d) implies the likely generation of donor–acceptor iron(ii) carbene intermediate.1c,^6^ Based on the obtained experimental results and mechanism study, we propose that the donor–acceptor diazo compound was decomposed by the bis(arylimino)pyridine iron(ii) catalyst to generate an iron(ii) carbene intermediate, which readily undergoes cyclopropanation of olefins to afford the cyclopropane product (Scheme 4b).

## Conclusions

In summary, the effective catalytic activity of bis(arylimino)pyridine iron(ii) complexes for carbene transfer reactions of donor–acceptor diazo compounds has been demonstrated by a range of metal carbene transformations from cyclopropanation and insertions to ylide formation. Notably, the asymmetric cyclopropanation of methyl phenyldiazoacetate and styrene has been achieved by a new chiral iron catalyst based on the bis(imino)pyridine ligand derivatized from l-valine methyl ester. Future studies will be aimed at developing new asymmetric bis(imino)pyridine iron catalysts for highly enantioselective metal carbene reactions, as well as elucidating the mechanism of such process and the nature of the iron carbene intermediate.

## Conflicts of interest

There are no conflicts to declare.

## Supplementary Material

Supplementary information
