# Supplementary material for: Bis(imino)pyridine iron complexes for catalytic carbene transfer reactions
Source: Chem Sci. 2019 Jul 2;10(34):7958–63. doi: 10.1039/c9sc02189b (PMC6839806; doi:10.1039/c9sc02189b)
Supplement: Supplementary file 1 [file SC-010-C9SC02189B-s001.pdf]

Electronic Supplementary Material (ESI) for Chemical Science.
This journal is © The Royal Society of Chemistry 2019

Bis(imino)pyridine Iron Complexes for Catalytic Carbene Transfer Reactions.

Ban Wang, Isaac G. Howard, Jackson W. Pope, Eric D. Conte, Yongming Deng*

Department of Chemistry, Western Kentucky University, Bowling Green, Kentucky 42101, United States

Corresponding Author
* E-mail: Yongming.deng@wku.edu

Supporting Information

Table of Contents

S1 **General Information and Materials**
S2 **General Procedures for the Synthesis of Achiral Bis(arylimino)pyridine Ligands and the Characterization Data.**
S3 **Procedures for the Synthesis of Chiral Bis(arylimino)pyridine Ligand (*S*)-^{VME}PDI and the Characterization Data.**
S4-5 **General Procedures for the Synthesis of Achiral Bis(alkylimino)pyridine Iron Complexes and the Characterization Data.**
S6 **Table S1. Screening of Iron Catalysts for Cyclopropanation.**
S7-11 **General Procedures for [(^{iPr}PDI)Fe($CH_3CN)_2$]($SbF_6)_2$ catalyzed Cyclopropanation of Diazo Compound 1 and Styrenes 2. Characterization Data of 3.**
S12 **Table S2. Screening of Iron Catalysts for Cyclopropanation.*[a]***
S13 **Procedures for the Chiral Bis(imino)pyridine Iron catalyzed Cyclopropanation of 1a and 2a. HPLC condition for 3a.**
S14 **Procedures for the Bis(arylimino)pyridine Iron catalyzed Epoxidation of 1a and Benzaldehyde. Characterization Data of 4.**
S15 **Procedures for the Bis(arylimino)pyridine Iron catalyzed Doyle–Kirmse Reaction of 1a and Allyl Phenyl Sulfide. Characterization Data of 5.**
S16 **Procedures for the Bis(arylimino)pyridine Iron catalyzed N–H insertion of 1a and Aniline. Characterization Data of 6.**
S16 **Procedures for the Bis(arylimino)pyridine Iron catalyzed C–H insertion of 1a and *N,N*-Dimethylaniline. Characterization Data of 7.**
S17-18 **General Procedures for the Bis(arylimino)pyridine Iron catalyzed O–H insertion of 1a and Alcohols/water. Characterization Data of 8.**
S19 **Mechanistic Study**
S19-20 **References**
S21-29 **^{1}H NMR and ^{13}C NMR Spectra of (*S*)-^{VME}PDI, 3a, 3p, 3q, 4, 5, 6, 7, and 8a.**
S30 **HPLC Spectra of 3a.**

General Information

All reactions, unless noted, were performed in oven-dried (150 °C) glassware with magnetic stirring. Analytical thin layer chromatography (TLC) was carried out using Merck KGaA silica gel 60 F254 plates; visualization was accomplished with UV light (254 nm). Liquid chromatography was performed using flash chromatography of the indicated system on silica gel (230-400 mesh). ^{1}H NMR spectra were recorded on a JEOL (500 MHz) spectrometer, and chemical shifts were reported in ppm. The peak information was described as: br = broad, s = singlet, d = doublet, t = triplet, q = quartet, m = multiplet, comp = composite; coupling constant(s) in Hz. The number of protons (n) for a given resonance was indicated by nH. Coupling constants were reported as J-values in Hz. ^{13}C NMR spectra were recorded on a JEOL (125 MHz) spectrometer with complete proton decoupling. Enantiomeric excesses were determined on a Varian 9020/9050 Series HPLC using Regis Whelk O-1 column. Melting points were obtained uncorrected from an Electro Thermo Mel-Temp DLX 100 device. High-resolution mass spectra (HRMS) were performed on a TOF-CS mass spectrometer using CsI as the standard. ESI-MS data were acquired using a Varian 500 Ion Trap LCMS spectrometer equipped with an ESI source and controlled by Varian workstation software.

Materials

Diazo compounds **1** were prepared according to the literature procedures, and their identities corresponded to those reported in the published literatures.[1] Metal salts, including $FeCl_2$ and $AgSbF_6$ were purchased from Strem Chemicals and used without further purification. All the other chemicals were obtained from commercial sources and used without further purification unless otherwise noted. The pyridine bis(oxazoline) ligands (PyBOX I and II) were purchased from Sigma-Aldrich. The oxazoline iminopyridine ligand (OIP) was prepared according to the literature procedures, and their identities corresponded to those reported in the published literatures.[2]

General Procedures for the Synthesis of Achiral Bis(arylimino)pyridine Ligands.

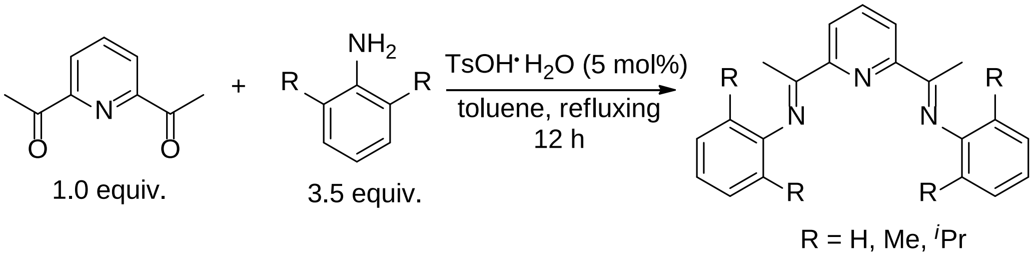

The achiral bis(arylimino)pyridine ligands were prepared according to the modified literature procedures.[3] 2,6-diacetylpyridine (3.5 g, 21.5 mmol, 1.0 equiv.) and arylamine (75.0 mmol, 3.5 equiv.) were dissolved in 75 mL of toluene in a round-bottom flask. *p*-Toluenesulfonic acid monohydrate (2 mol%, 0.43 mmol, 74.0 mg) were added, and the solution was refluxed for 12 hours. Then the reaction solution was concentrated under reduced pressure resulting yellow solid. The yellow solid was recrystallized by hot ethanol to give a yellow solid. Analytical data for these products is listed below.

2,6-Bis-[1-(2,6-dimethylphenylimino)ethyl]pyridine.

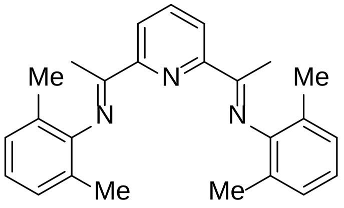

Yellow solid, 66% yield, 5.21 g. ^{1}H NMR (500 MHz, $CDCl_3$) δ 8.50 (d, *J* = 7.8 Hz, 2H), 7.89 (t, *J* = 7.8 Hz, 1H), 7.13 – 6.98 (comp, 6H), 2.27 (s, 6H), 2.06 (s, 12H). ^{13}C NMR (126 MHz, $CDCl_3$) δ 167.31, 155.28, 149.06, 137.12, 128.08, 125.57, 123.12, 122.58, 18.21, 16.74. This compound has been previously reported; the spectroscopic data are identical to those in reference.

2,6-bis-[1-(2,6-diisopropylphenylimino)ethyl]pyridine.

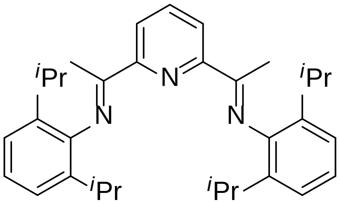

Yellow solid, 62% yield, 6.42 g. ^{1}H NMR (500 MHz, $CDCl_3$) δ 8.58 (d, *J* = 7.8 Hz, 2H), 7.92 (t, *J* = 7.8 Hz, 1H), 7.20 – 7.10 (comp, 6H), 2.78 (sept, *J* = 6.8 Hz, 4H), 2.30 (s, 6 H,), 1.18 (dd, *J* = 6.8 Hz, 24H). ^{13}C NMR (126 MHz, $CDCl_3$) δ 167.24, 155.25, 146.48, 135.87, 123.75, 123.24, 122.67, 122.41, 28.39, 23.38, 17.17. This compound has been previously reported; the spectroscopic data are identical to those in reference.

Procedures for the Synthesis of Chiral Bis(arylimino)pyridine Ligand (*S*)-^{VME}PDI.

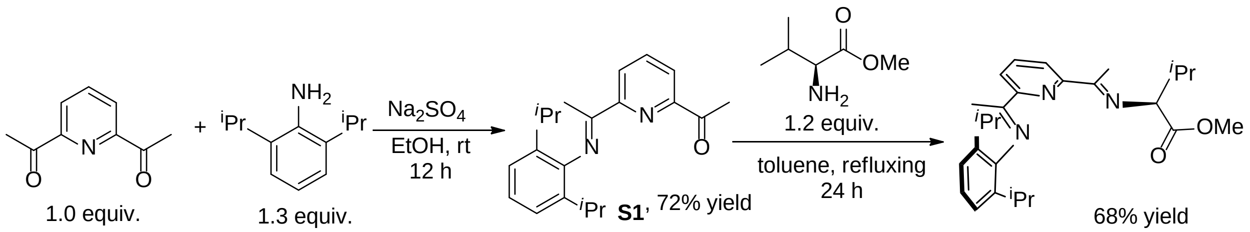

2,6-Diacetylpyridine (1.63 g, 10.0 mmol, 1.0 equiv.) and 2,6-diisopropylaniline (2.30 g, 13 mmol, 1.3 equiv.) were dissolved in 50 mL of ethanol in a round-bottom flask. Anhydrous sodium sulfate (6 g) were added, and the solution was stirred for 12 hours. Then the reaction solution was filtered to remove sodium sulfate. The filtrate was concentrated under reduced pressure resulting yellow solid, which was washed twice by cold ethanol. The solid was then recrystallized by hot ethanol to give off-white solid product **S1** and dried under vacuum (72% yield, 2.32 g).

Enantiopure *L*-Valine methyl ester (472.0 mg, 3.6 mmol, 1.2 equiv.) and the single-sided ligand **S1** (967.2 mg, 3 mmol, 1.0 equiv.) were dissolved in 40 mL of toluene in a round-bottom flask. The reaction was set to reflux, and the water was removed using a Dean-Stark apparatus containing sodium sulfate. After 24 hours, the solvent was removed under reduced pressure and the yellowish residue was recrystallized from a minimal amount of hot ethanol. The off-white solid was collected by filtration, washed with cold (-20 °C) ethanol (10 mL) and dried under vacuum (68% yield, 887.5 mg). Analytical data for (*S*)-^{VME}PDI is listed below.

(S)-Methyl 2-((1-(6-(1-((2,6-diisopropylphenyl)imino)ethyl)pyridin-2-yl)ethylidene)amino)-3-methylbutanoate (*S*)-^{VME}PDI.

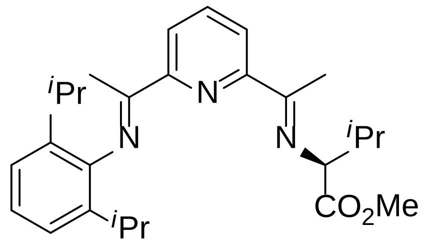

Off-white solid, 68% yield, 887.5 mg, m.p. 118 – 120 °C. $[\alpha]_D^{22}$ = +143.2 (c = 1.20, chloroform). ^{1}H NMR (500 MHz, CHLOROFORM-D) δ 8.37 (dd, *J* = 7.8, 1.0 Hz, 1H), 8.32 (dd, *J* = 7.8, 1.0 Hz, 1H), 7.83 (t, *J* = 7.8 Hz, 1H), 7.18 – 7.15 (comp, 2H), 7.11 – 7.08 (m, 1H), 4.18 (d, *J* = 6.8 Hz, 1H), 3.74 (s, 3H), 2.77 – 2.71 (comp, 2H), 2.45 (s, 3H), 2.48 – 2.41 (comp, 4H), 1.14 (d, *J* = 6.9 Hz, 12H), 1.01 (dd, *J* = 6.8, 0.6 Hz, 6H). ^{13}C NMR (126 MHz, CHLOROFORM-D) δ 172.41, 168.99, 167.08, 156.22, 154.87, 146.61, 136.85, 135.92, 135.88, 123.64, 123.10, 123.07, 122.60, 121.93, 70.79, 52.02, 32.63, 28.41, 28.38, 23.32, 23.30, 23.03, 23.01, 19.67, 18.72, 17.28, 14.44. HRMS

(ESI) calculated for $C_{27}H_{37}N_3O_2Cs$ $[M+Cs]^+$: 568.1940; found: 568.1947.

General Procedures for the Synthesis of Achiral Bis(alkylimino)pyridine Iron Complexes.

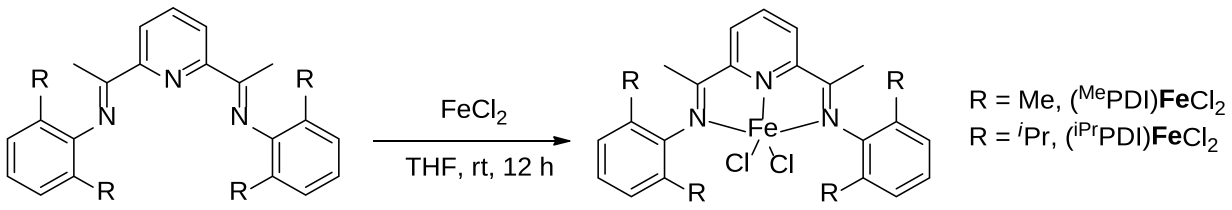

The achiral bis(arylimino)pyridine iron complexes, (^{Me}PDI)$FeCl_2$ and (^{iPr}PDI)$FeCl_2$, were prepared according to the literature procedures.[3,4] A Schlenk flask was charged with 1.0 mmol of achiral bis(arylimino)pyridine ligand, 1.0 mmol of $FeCl_2$, and 25 mL of THF under nitrogen protection. The reaction mixture was stirred 12 hours. Diethyl ether was then added to the reaction to precipitate the complex, and the resultant solids were filtered in air and washed with Et_2O and pentane and dried in vacuo.

(^{Me}PDI)$FeCl_2$

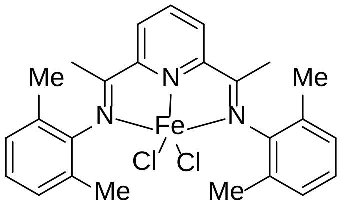

Blue solid, 74% yield, 297.2 mg. ^1H NMR (CD_2Cl_2, broad singlets are observed in each case): δ 81.0 (2H), 45.3 (1H), 17.6 (4H), 15.8 (12H), -10.6 (2H), -19.2 (6H). This compound has been previously reported; the spectroscopic data are identical to those in reference.[4a]

(^{iPr}PDI)$FeCl_2$

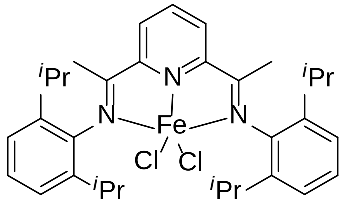

Blue solid, 71% yield, 510.1 mg. ^1H NMR (CD_2Cl_2, broad singlets are observed in each case): δ 87.0 (2H), 77.5 (1H), 16.5 (4H), -4.5 (24H), -8.9 (2H), -20.2 (4H), -33.8 (6H). This compound has been previously reported; the spectroscopic data are identical to those in reference.[3b]

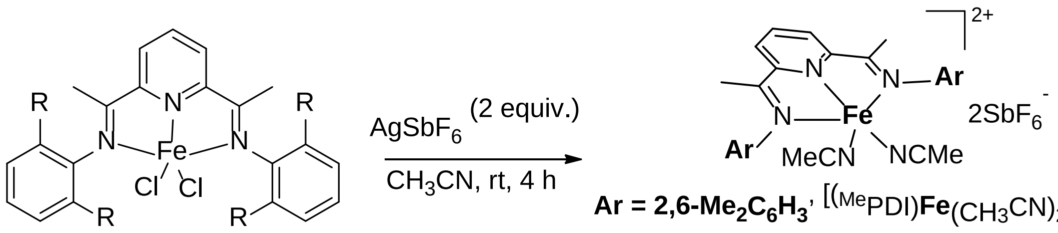

Ar = 2,6-Me$_2$C$_6$H$_3$, [(^{Me}PDI)**Fe**(CH$_3$CN)$_2$](SbF$_6$)$_2$

Ar = 2,6-^{i}Pr$_2$C$_6$H$_3$, [(^{iPr}PDI)**Fe**(CH$_3$CN)$_2$](SbF$_6$)$_2$

The achiral bis(arylimino)pyridine iron complexes, [(^{Me}PDI)Fe(CH$_3$CN)$_2$](SbF$_6$)$_2$ and [(^{iPr}PDI)Fe(CH$_3$CN)$_2$](SbF$_6$)$_2$, were prepared according to the literature procedures.[5] A Schlenk flask was charged with 0.60 mmol of (^{Me}PDI)FeCl$_2$ or (^{iPr}PDI)FeCl$_2$, 2 equivalents (412 mg, 1.20 mmol) of silver(I) hexafluoroantimonate, AgSbF$_6$, and 25 mL of acetonitrile (CH$_3$CN) under nitrogen protection. The solution was stirred for 4 hours at room temperature and subsequently reduced to dryness. Then 10 ml of dichloromethane was added and the solid AgCl was filtered off. The filtrate was concentrated under reduced pressure to about 5 mL, and pentane was added to precipitate an orange color solid. This solid was washed once with pentane and twice with diethyl ether to give the product as an orange powder.

[(^{Me}PDI)Fe(CH$_3$CN)$_2$](SbF$_6$)$_2$

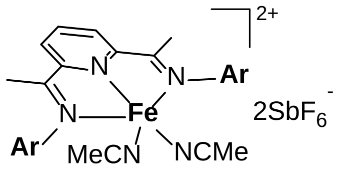

Ar = 2,6-Me$_2$C$_6$H$_3$

Orange solid, 63% yield, 370.1 mg. ^{1}H NMR (CD$_2$Cl$_2$, broad singlets are observed in each case): δ 72.3 (2H), 50.1 (6H, C*H$_3$*CN), 28.7 (1H), 15.4 (12H), 13.2 (4H), 4.7 (6H), −15.1 (2H). This compound has been previously reported; the spectroscopic data are identical to those in reference.[5]

[(^{iPr}PDI)Fe(CH$_3$CN)$_2$](SbF$_6$)$_2$

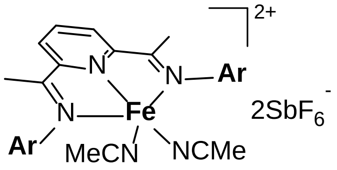

Ar = 2,6-^{i}Pr$_2$C$_6$H$_3$

Orange solid, 66% yield, 431.8 mg. ^{1}H NMR (CD$_2$Cl$_2$, broad singlets are observed in each case): δ 94.3 (2H), 56.6 (6H, C*H$_3$*CN), 34.4 (6H), 14.3 (4H), 9.7 (1H), 5.4 (12H), 4.3 (12 H), −16.9 (2H). This compound has been previously reported; the spectroscopic data are identical to those in reference.[5]

Table S1. Screening of Iron Catalysts for Cyclopropanation.[a]

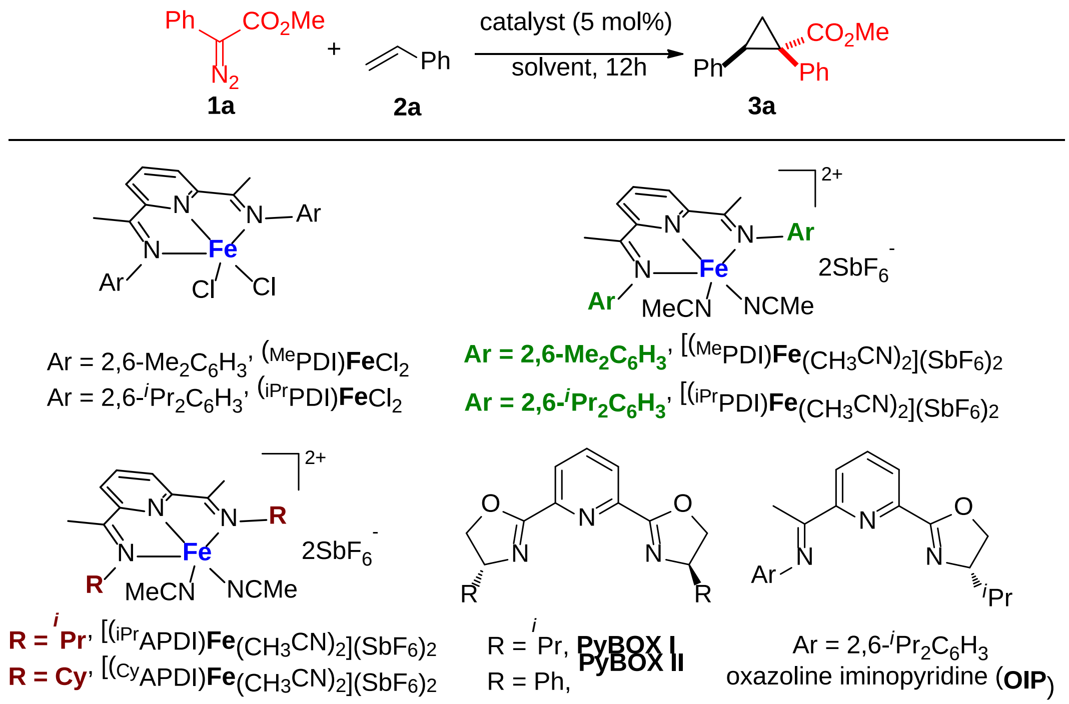

entry	catalyst	solvent	T [°C]	yield (%)[b]
1	(^{Me}PDI)$FeCl_2$	DCE	50	14
2	(^{iPr}PDI)$FeCl_2$	DCE	50	18
3	[(^{Me}PDI)Fe$(CH_3CN)_2$]$(SbF_6)_2$	DCE	rt	65
**4**	**[(^{iPr}PDI)Fe$(CH_3CN)_2$]$(SbF_6)_2$**	DCE	rt	**86**
5	(^{iPr}PDI)$FeCl_2$/NaBArF (10 mol%)	DCE	rt	48
6	[(^{iPr}PDI)Fe$(CH_3CN)_2$]$(SbF_6)_2$	DCM	rt	68
7	[(^{iPr}PDI)Fe$(CH_3CN)_2$]$(SbF_6)_2$	toluene	rt	58
8	[(^{iPr}PDI)Fe$(CH_3CN)_2$]$(SbF_6)_2$	chlorobenzene	rt	71
9	[(^{iPr}PDI)Fe$(CH_3CN)_2$]$(SbF_6)_2$	diethyl ether	rt	34
10	[(^{iPr}PDI)Fe$(CH_3CN)_2$]$(SbF_6)_2$	CH_3CN	rt	8
11	[(^{iPr}APDI)Fe$(CH_3CN)_2$]$(SbF_6)_2$	DCE	50	8
12	[(^{Cy}APDI)Fe$(CH_3CN)_2$]$(SbF_6)_2$	DCE	50	< 5
13	$FeCl_2$/PyBOX(I)/$AgSbF_6$	DCE	50	< 5
14	$FeCl_2$/PyBOX(II)/$AgSbF_6$	DCE	50	< 5
15	$FeCl_2$/OIP/ $AgSbF_6$		50	9

[a]Reaction condition unless otherwise noted: **1a** (0.20 mmol, 1.0 equiv.) in dry DCE (1.0 ml) was added to a 1.0 mL DCE solution of **2a** (1.0 mmol, 5.0 equiv.), and catalyst (0.01 mmol) under N_2 within 1 hour. [b]Yield of isolated product **3a** based on the limiting reagent **1a**. (1,2-dichloroethane = DCE, NaBArF = sodium tetrakis[3,5-bis(trifluoromethyl)phenyl]borate, CH_3CN = acetonitrile, DCM = dichloromethane)

General Procedures for the Bis(arylimino)pyridine Iron Complex, [(^{iPr}PDI)Fe(CH_3CN)$_2$](SbF_6)$_2$, catalyzed Cyclopropanation of Diazo Compound 1 and Styrenes 2.

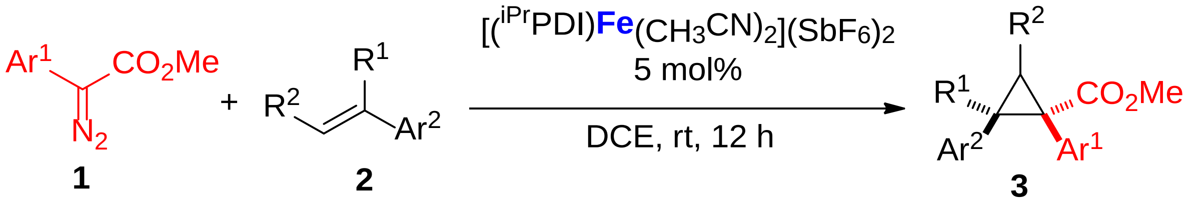

To a two-dram vial containing a magnetic stirring bar, [(^{iPr}PDI)Fe(CH_3CN)$_2$](SbF_6)$_2$ (21.8 mg, 0.020 mmol, 5 mol%) and 2.0 mL of dry 1,2-dichloroethane (DCE) were added sequentially under a nitrogen atmosphere. Then styrene **2** (2.0 mmol, 5.0 equiv.) was subjected into the system. The flask was capped by a rubber septum, and its solution was stirred before diazo compound **1** (0.40 mmol, 1.0 equiv.) dissolved in 1.0 mL of dry DCE was added. Stirring was continued at room temperature until all the diazo compound **1** was consumed based on TLC analysis. Subsequently, the reaction mixture was passed through a short pad of Celite and concentrated under reduced pressure. The diastereomeric ratio was determined by ^{1}H NMR spectroscopy of the residue, which was then purified by flash chromatography on silica gel using 10:1 hexanes:ethyl acetate as the eluent to afford the corresponding cyclopropanation product **3**. Analytical data for these products is listed below.

(±) (1S,2R)-Methyl 1,2-diphenylcyclopropanecarboxylate 3a.

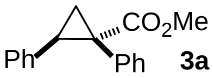

3a

White solid, 86% yield, 86.5 mg, m.p. 59.0 – 61.1 °C, dr > 20:1. ^{1}H NMR (500 MHz, $CDCl_3$) δ 7.15 – 7.11 (comp, 3H), 7.09 – 6.97 (comp, 5H), 6.77 – 6.74 (comp, 2H), 3.67 (s, 3H), 3.12 (dd, *J* = 9.4, 7.3 Hz, 1H), 2.14 (dd, *J* = 9.4, 4.9 Hz, 1H), 1.89 (dd, *J* = 7.3, 4.9 Hz, 1H). ^{13}C NMR (126 MHz, $CDCl_3$) δ 174.47, 136.43, 134.79, 132.02, 128.12, 127.78, 127.77, 127.12, 126.39, 52.75, 37.48, 33.23, 20.61. HRMS (ESI) calculated for $C_{17}H_{16}O_2Cs$ $[M+Cs]^+$: 385.0205; found: 385.0209. The spectroscopic date and stereochemical assignment is consistent with previously reported results.[6]

(±) (1S,2R)-Methyl 2-phenyl-1-(p-tolyl)cyclopropanecarboxylate 3b.

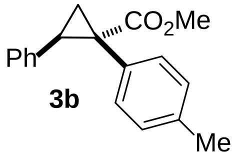

3b

White solid, 81% yield, 86.1 mg, dr > 20:1. ^{1}H NMR (500 MHz, $CDCl_3$) δ 7.11 – 7.07 (comp, 3H), 6.96 – 6.89 (comp, 4H), 6.81 – 6.77 (comp, 2H), 3.67 (s, 3H), 3.11 (dd, *J* = 9.4, 7.2 Hz, 1H), 2.27 (s, 3H), 2.14 (dd, *J* = 9.4, 5.0 Hz, 1H),1.90 (dd, *J* = 7.2, 5.0 Hz, 1H). ^{13}C NMR (126 MHz, $CDCl_3$) δ 174.57, 136.51, 136.42, 131.72, 131.63, 128.75, 128.38, 127.94, 126.57, 52.81, 37.24, 33.46, 21.32, 20.67. The spectroscopic date and stereochemical assignment is consistent with previously reported results.[7]

(±) (1S,2R)-Methyl 1-(4-methoxyphenyl)-2-phenylcyclopropanecarboxylate 3c.

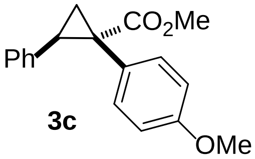

3c

Colorless oil, 83% yield, 93.4 mg, dr > 20:1. ^{1}H NMR (500 MHz, $CDCl_3$) δ 7.13 – 7.09 (comp, 3H), 6.99 – 6.95 (comp, 2H), 6.84 – 6.80 (comp, 2H), 6.73 – 6.68 (comp, 2H), 3.75 (s, 3H), 3.70 (s, 3H), 3.14 (dd, *J* = 9.0, 7.4 Hz, 1H), 2.19 – 2.15 (dd, *J* = 9.0, 5.0 Hz, 1H), 1.89 - 1.86 (dd, *J* = 7.4, 5.0 Hz, 1H). ^{13}C NMR (126 MHz, $CDCl_3$) δ 174.69, 158.48, 136.46, 132.72, 128.05, 127.58, 126.64, 126.18, 113.09, 54.96, 52.60, 36.67, 33.13, 20.68. The spectroscopic date and stereochemical assignment is consistent with previously reported results.[7]

(±) (1S,2R)-Methyl 1-(4-chlorophenyl)-2-phenylcyclopropanecarboxylate 3d.

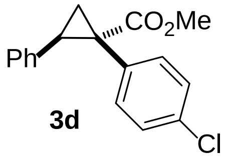

3d

White solid, 88% yield, 100.4 mg, dr > 20:1. ^{1}H NMR (500 MHz, $CDCl_3$) δ 7.14 – 7.06 (comp, 5H), 6.96 (*J* = 8.7 Hz, 2H), 6.79 – 6.76 (comp, 2H), 3.68 (s, 3H), 3.12 (dd, *J* = 9.1, 7.4 Hz, 1H), 2.14 (dd, *J* = 9.1, 5.0, 1H), 1.88 (dd, *J* = 7.4, 5.0 Hz, 1H). ^{13}C NMR (126 MHz, $CDCl_3$) δ 174.15, 136.50, 133.83, 133.57, 133.31, 128.27, 128.16, 128.10, 126.83, 52.95, 37.01, 33.41, 20.8. The spectroscopic date and stereochemical assignment is consistent with previously reported results.[8]

(±) (1S,2R)-Methyl 1-(4-bromophenyl)-2-phenylcyclopropanecarboxylate 3e.

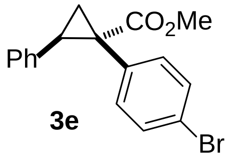

3e

White solid, 83% yield, 110.1 mg, dr > 20:1. ^{1}H NMR (500 MHz, $CDCl_3$) δ 7.26 (d, *J* = 8.6 Hz, 2H), 7.11 – 7.07 (comp, 3H), 6.90 (d, *J* = 8.6 Hz, 2H), 6.79 – 6.75 (comp, 2H), 3.65 (s, 3H), 3.14 (dd, *J* = 9.0, 7.3 Hz, 1H), 2.13 (dd, *J* = 9.0, 5.0 Hz, 1H), 1.84 (dd, *J* = 7.3, 5.0 Hz, 1H). ^{13}C NMR (126 MHz, $CDCl_3$) δ 173.84, 139.00, 135.87, 133.57, 130.89, 128.01, 127.84, 126.45, 121.12, 52.67, 36.96, 33.06, 20.21. The spectroscopic date and stereochemical assignment is consistent with previously

reported results.[7]

(±) (1S,2R)-Methyl 1-(naphthalen-2-yl)-2-phenylcyclopropanecarboxylate 3f.

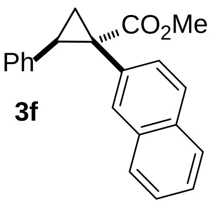

3f

White solid, 81% yield, 97.0 mg, dr = 18:1. ^{1}H NMR (500 MHz, $CDCl_3$) δ 7.72 – 7.68 (comp, 2H), 7.60 (s, 1H), 7.54 (d, *J* = 8.5 Hz, 1H), 7.42 – 7.38 (comp, 2H), 7.06 – 7.04 (m, 1H), 7.01 – 6.98 (comp, 3H), 6.83 – 6.81 (comp, 2H), 3.64 (s, 3H), 3.18 (dd, *J* = 8.9, 7.5 Hz, 1H), 2.21 (dd, *J* = 8.9, 5.0 Hz, 1H), 2.00 (dd, *J* = 7.5, 5.0 Hz, 1H). ^{13}C NMR (126 MHz, $CDCl_3$) δ 174.81, 136.53, 133.37, 133.0, 132.72, 130.92, 130.43, 128.38, 128.19, 128.00, 127.92, 127.40, 126.68, 126.09, 125.92, 52.93, 37.84, 33.56, 20.79. The spectroscopic date and stereochemical assignment is consistent with previously reported results.[7]

(±) (1S,2R)-methyl 1-(2-methoxyphenyl)-2-phenylcyclopropanecarboxylate 3h.

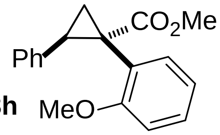

3h

Light yelow oil, 52% yield, 58.8 mg, dr = 15:1. ^{1}H NMR (500 MHz, $CDCl_3$) δ 7.20 – 7.12 (comp, 2H), 7.06 – 6.99 (comp, 3H), 6.89 – 6.88 (m, 1H), 6.82 – 6.76 (comp, 2H), 6.53 (d, J = 8.1 Hz, 1H), 3.66 (s, 3H), 3.35 (s, 3H), 3.25 (dd, J = 9.5, 7.8 Hz, 1H), 2.00 (dd, J = 9.5, 4.8 Hz, 1H), 1.86 (dd, J = 7.8 and 4.8 Hz, 1H). ^{13}C NMR (126 MHz, $CDCl_3$) δ 174.68, 159.21, 137.05, 131.78, 128.84, 127.93, 127.29, 126.08, 124.12, 120.08, 110.52, 55.21, 52.68, 34.32, 32.59, 20.81. The spectroscopic date and stereochemical assignment is consistent with previously reported results.[8]

(±) (1S,2R)-methyl 1-(2-chlorophenyl)-2-phenylcyclopropanecarboxylate 3i.

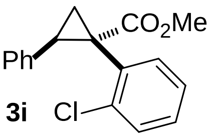

3i

White solid, 58% yield, 66.3 mg, dr > 20:1. ^{1}H NMR (500 MHz, $CDCl_3$) δ 7.19 – 7.04 (comp, 7H), 6.85 – 6.78 (comp, 2H), 3.69 (s, 3H), 3.14 (dd, *J* = 8.8, 7.2 Hz, 1H), 2.12 (dd, *J* = 8.8, 5.1, 1H), 1.93 (dd, *J* = 7.2, 5.1 Hz, 1H). ^{13}C NMR (126 MHz, $CDCl_3$) δ 173.58, 137.47, 133.53, 129.67, 129.0, 128.17, 127.56, 127.43, 126.73, 126.44, 124.66, 52.93, 33.60, 30.01, 21.88. The spectroscopic date and stereochemical assignment is consistent with previously reported results.[8]

(±) (1S,2R)-Methyl 1-phenyl-2-(p-tolyl)cyclopropanecarboxylate 3j.

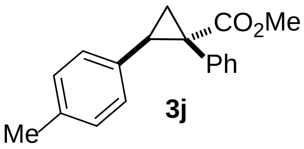

Colorless oil, 91% yield, 96.3 mg, dr > 20:1. ^{1}H NMR (500 MHz, $CDCl_3$) δ 7.15 – 7.11 (comp, 3H), 7.08 – 7.05 (comp, 2H), 6.85 (d, J = 8.0 Hz, 2H), 6.64 (d, J = 8.0 Hz, 2H), 3.64 (s, 3H), 3.06 (dd, J = 9.2, 6.8 Hz, 1H), 2.23 (s, 3H), 2.14 (dd, J = 9.2, 4.8 Hz, 1H), 1.86 (dd, J = 6.8, 4.8, 1H). ^{13}C NMR (126 MHz, $CDCl_3$) δ 174.68, 135.97, 135.00, 133.72, 132.34, 129.05, 128.13, 127.86, 127.05, 53.14, 37.83, 34.17, 21.08, 20.60. The spectroscopic date and stereochemical assignment is consistent with previously reported results.[9]

(±) (1S,2R)-Methyl 2-(4-methoxyphenyl)-1-phenylcyclopropanecarboxylate 3k.

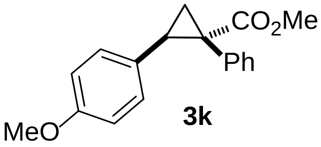

Light yellow solid, 88% yield, 99.6 mg, dr > 20:1. ^{1}H NMR (500 MHz, $CDCl_3$) δ ^{13}C NMR (126 MHz, $CDCl_3$) δ 7.20 – 7.14 (comp, 3H), 7.09 – 7.03 (comp, 2H), 6.78 – 6.65 (comp, 4H), 3.69 (s, 3H), 3.63 (s, 3H), 3.05 (dd, J = 10.0, 8.8 Hz, 1H), 2.10 (dd, J = 10.0, 4.6 Hz, 1H), 1.80 (dd, J = 8.8, 4.6 Hz, 1H). 174.63, 158.21, 134.93, 132.00, 129.04, 128.42, 127.74, 126.98, 113.41, 55.20, 52.61, 37.10, 32.74, 20.61. The spectroscopic date and stereochemical assignment is consistent with previously reported results.[9]

(±) (1S,2R)-Methyl 2-(4-chlorophenyl)-1-phenylcyclopropanecarboxylate 3l.

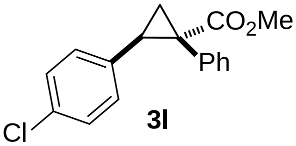

White solid, 90% yield, 103.1 mg, dr > 20:1. ^{1}H NMR (500 MHz, $CDCl_3$) δ 7.14 – 7.10 (comp, 3H), 7.06 – 7.02 (comp, 4H), 6.72 – 6.69(comp, 2H), 3.68 (s, 3H), 3.10 (dd, J = 9.0, 7.0 Hz, 1H), 2.14 (dd, J = 9.0, 4.9 Hz, 1H), 1.85 (dd, J = 7.0, 4.9 1H). ^{13}C NMR (126 MHz, $CDCl_3$) δ 175.43, 158.98, 136.15, 134.10, 132.45, 130.25, 128.45, 126.59, 114.00, 55.88, 53.08, 37.01, 32.95, 21.32. The spectroscopic date and stereochemical assignment is consistent with previously reported results.[9]

(±) (1S,2R)-Methyl 1-phenyl-2-(4-(trifluoromethyl)phenyl)cyclopropanecarboxylate 3m.

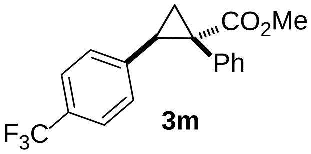

White solid, 67% yield, 85.0 mg, dr > 20:1. ^{1}H NMR (500 MHz, $CDCl_3$) δ 7.30 (d, J = 8.0 Hz, 2H), 7.21 – 7.12 (comp, 3H), 7.08 – 6.92 (comp, 2H), 6.87 (d, J = 8.0 Hz, 2H), 3.68 (s, 3H), 3.16 (dd, J = 9.0, 6.5 Hz, 1H), 2.21 (dd, J = 9.0, 4.8 Hz, 1H), 1.94 (dd, J = 6.5, 4.8, 1H). ^{13}C NMR (126 MHz, $CDCl_3$) δ 175.34, 159.68, 141.73 (q, J = 2.0 Hz), 133.54, 130.10 (q, J = 34.5 Hz), 129.90, 126.81, 126.21 (q, J = 5.1 Hz), 125.00 (q, J = 281.2 Hz), 114.45, 55.80, 53.68, 37.91, 33.58, 21.77. The

spectroscopic date and stereochemical assignment is consistent with previously reported results.[9]

Methyl 1,2,2-triphenylcyclopropanecarboxylate 3n.

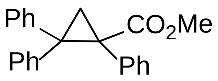

3n

White solid, 73% yield, 95.5 mg. ^{1}H NMR (500 MHz, $CDCl_3$) δ 7.46 – 7.41 (comp, 2H), 7.31 – 7.22 (comp, 5H), 7.12 – 7.10 (comp, 3H), 6.92 – 6.86 (comp, 5H), 3.68 (s, 3H), 2.70 (d, J = 5.5 Hz, 1H), 2.54 (d, J = 5.5 Hz, 1H). ^{13}C NMR (126 MHz, $CDCl_3$) δ 172.02, 142.51, 139.72, 135.92, 131.99, 130.12, 128.86, 128.43, 127.51, 126.98, 126.12, 52.43, 44.54, 43.54, 23.00. The spectroscopic date and stereochemical assignment is consistent with previously reported results.[6]

(±) (1S,3R)-Methyl 2-methyl-1,3-diphenylcyclopropanecarboxylate 3o.

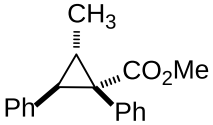

3o

White solid, 70% yield, 74.8 mg, dr = 17:1. ^{1}H NMR (500 MHz, $CDCl_3$) δ 7.15 – 7.12 (comp, 3H), 7.08 – 6.99 (comp, 5H), 6.80 – 6.75 (comp, 2H), 3.66 (s, 3H), 3.10 (d, J = 8.0, 1H), 2.30 – 2.26 (comp, 1H), 1.48 (d, J = 6.8, 3H). ^{13}C NMR (126 MHz, $CDCl_3$) δ 173.01, 138.21, 137.02, 132.42, 128.10, 127.91, 127.58, 127.01, 126.25, 53.10, 42.65, 37.94, 27.99, 13.00. The spectroscopic date and stereochemical assignment is consistent with previously reported results.[6]

Methyl 7-phenylbicyclo[4.1.0]hept-2-ene-7-carboxylate 3p.

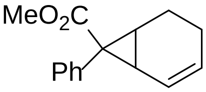

3p

White solid, 80% yield, 73.1 mg, dr > 20:1. ^{1}H NMR (500 MHz, $CDCl_3$) δ 7.27 – 7.25 (comp, 3H), 7.22 – 7.20 (comp, 2H), 6.03 – 5.97 (m, 1H), 5.41 (ddd, J = 10.0, 5.9, 2.5 Hz, 1H), 3.56 (s, 3H), 2.36 – 2.27 (comp, 2H), 2.00 – 1.93 (m, 1H), 1.86 – 1.74 (m, 1H), 1.60 – 1.53 (m, 1H), 0.45 – 0.35 (m, 1H). ^{13}C NMR (126 MHz, $CDCl_3$) δ 174.15, 134.79, 131.78, 128.56, 127.84, 127.02, 122.74, 52.57, 52.56, 40.41, 27.82, 27.81, 25.74, 21.09, 16.84. HRMS (ESI) calculated for $C_{15}H_{16}O_2Cs$ $[M+Cs]^+$: 361.0205; found: 361.0206. The spectroscopic date and stereochemical assignment is consistent with previously reported results.[6]

Methyl 7-phenylbicyclo[4.1.0]heptane-7-carboxylate 3q.

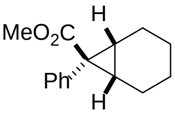

3q

White solid, 72% yield, 66.4 mg, dr > 20:1. ^{1}H NMR (500 MHz, $CDCl_3$) δ 7.37 – 7.29 (comp, 3H), 7.27 – 7.24 (comp, 2H), 3.55 (s, 3H), 2.05 – 1.94 (comp, 4H), 1.77 – 1.71 (comp, 2H), 1.10 – 1.04 (comp, 2H), 0.62 – 0.59 (m, 2H). ^{13}C NMR (126 MHz, $CDCl_3$) δ 175.85, 134.80, 132.33, 128.36, 126.79, 52.43, 52.56, 26.02, 20.98, 20.34. HRMS (ESI) calculated for $C_{15}H_{19}O_2$ $[M+H]^+$: 331.1385; found: 331.1390. The spectroscopic date

and stereochemical assignment is consistent with previously reported results.[6]

(±) (1R,2R)-methyl 2-phenyl-1-((E)-styryl)cyclopropanecarboxylate 3r.

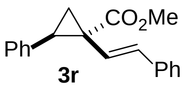

Colorless oil, 84% yield, 93.6 mg, dr > 20:1. ^{1}H NMR (500 MHz, $CDCl_3$) δ 7.25 – 7.20 (comp, 4H), 7.19 – 7.10 (comp, 6H), 6.35 (d, J = 16.0 Hz, 1H), 6.13 (d, J = 16.0 Hz, 1H), 3.76 (s, 3H), 3.01 (dd, J = 9.0, 7.4 Hz, 1H), 2.02 (dd, J = 9.2, 5.0 Hz, 1H), 1.83 (dd, J = 7.4, 5.0 Hz, 1H). ^{13}C NMR (126 MHz, $CDCl_3$) δ 174.26, 137.16, 135.60, 133.17, 129.19, 128.48, 128.09, 127.44, 126.87, 126.34, 124.18, 52.55, 35.07, 33.39, 18.72. HRMS (ESI) calculated for $C_{19}H_{18}O_2Cs$ $[M+Cs]^+$: 411.0361; found: 411.0365. The spectroscopic date and stereochemical assignment is consistent with previously reported results.[6]

Methyl 1,2-diphenylcycloprop-2-enecarboxylate 3s.

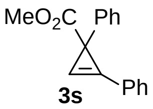

Colorless oil, 61% yield, 61.3 mg. ^{1}H NMR (500 MHz, $CDCl_3$) δ 7.70 – 7.61 (comp, 2H), 7.480 – 7.37 (comp, 5H), 7.30 – 7.25 (comp, 2H), 7.21 – 7.10 (comp, 2H), 3.72 (s, 3H). ^{13}C NMR (126 MHz, $CDCl_3$) δ 175.12, 140.93, 123.00, 129.89, 128.84, 128.09, 128.23, 126.45, 125.53, 117.34, 100.41, 52.54, 33.83. HRMS (ESI) calculated for $C_{19}H_{18}O_2Cs$ $[M+Cs]^+$: 411.0361; found: 411.0365. The spectroscopic date is consistent with previously reported results.[10]

Table S2. Screening of Iron Catalysts for Cyclopropanation.[a]

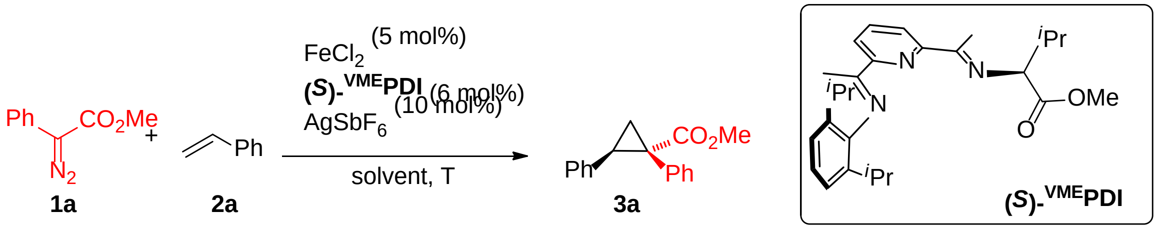

entry	solvent	T [°C]	yield (%)[b]	ee (%)[c]
**1**	**DCE**	**rt**	**78**	**65**
2	DCE	40	89	34
3[d]	DCE	0	42	77
4	DCM	rt	69	**58**
5	toluene	rt	72	21
6	diethyl ether	rt	32	48
7	THF	rt	< 5	-
8	pentane	rt	43	63

9	CH_3CN	rt	< 5	-

[a]Reaction condition unless otherwise noted: Reaction conditions: **1a** (0.20 mmol, 1.0 equiv.) in dry solvent (1.0 mL) was added to a 1.0 mL solution of **2a** (1.0 mmol, 5.0 equiv.), ligand (0.024 mmol), $FeCl_2$ (0.01 mmol), and $AgSbF_6$ (0.04 mmol) under N_2 within 1 h. [b]Yield of isolated product **3a** based on the limiting reagent **1a**. [c]Enantiomeric excesses of **3a** were determined by chiral HPLC analysis. [d]24 hours. (1,2-dichloroethane = DCE, THF = tetrahydrofuran, CH_3CN = acetonitrile, DCM = dichloromethane).

Procedures for the Chiral Bis(imino)pyridine Iron catalyzed Cyclopropanation of 1a and 2a.

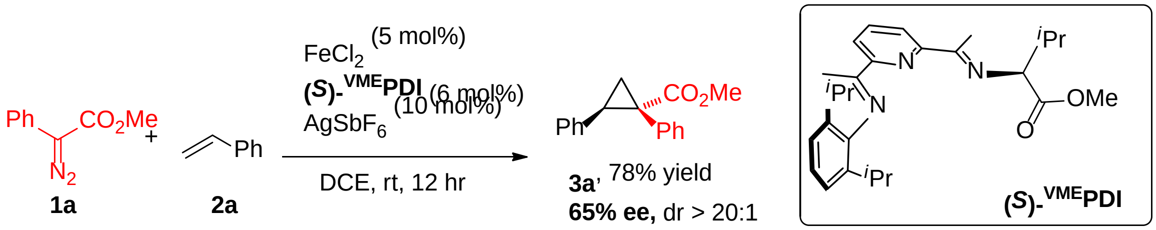

To a two-dram vial containing a magnetic stirring bar, $FeCl_2$ (2.5 mg, 0.020 mmol, 5 mol%), chiral bis(imino)pyridine ligand **(*S*)-^{VME}PDI** (10.6 mg, 0.024 mmol, 6 mol%) and 2.0 mL of dry 1,2-dichloroethane (DCE) were added sequentially under a nitrogen atmosphere. The resulting mixture was stirred at room temperature under nitrogen atmosphere for 1 h, which turned from colorless to blue. Then $AgSbF_6$ (13.7 mg, 0.040 mmol, 10 mol%) in 1.0 mL of dry 1,2-dichloroethane (DCE) was injected to the reaction that was stirred at room temperature under nitrogen atmosphere for another 1 h. The color of the reaction mixture turned from blue to orange. Next, styrene **2a** (208.0 mg, 2.0 mmol, 5.0 equiv.) was subjected into the system. The flask was capped by a rubber septum, and its solution was stirred before diazo compound **1a** (70.5 mg, 0.40 mmol, 1.0 equiv.) dissolved in 1.0 mL of dry DCE was added via syringe pump over 1 h. Stirring was continued at room temperature until all the diazo compound **1a** was consumed based on TLC analysis. Subsequently, the reaction mixture was passed through a short pad of Celite and concentrated under reduced pressure. The diastereomeric ratio was determined by ^{1}H NMR spectroscopy of the residue, which was then purified by flash chromatography on silica gel using 10:1 hexanes:ethyl acetate as the eluent to afford the corresponding cyclopropanation product **3a** in 78% yield, 78.7 mg.

Enantiomeric excesses of **3a** (65% ee) were determined on a Varian 9020/9050 Series HPLC using Regis Whelk O-1 column. HPLC conditions for determination of enantiomeric excess: Regis Whelk O-1 column, 254 nm, hexanes/*i*-PrOH = 98:2, 1.0 mL/min, t_r (major) = 12.24 min, t_r (minor)

= 10.98 min. $[\alpha]_D^{22}$ = +27.8 (c = 1.84, chloroform). The spectroscopic date and stereochemical assignment is consistent with previously reported results.[7]

Procedures for the Bis(arylimino)pyridine Iron catalyzed Epoxidation of 1a and Benzaldehyde.

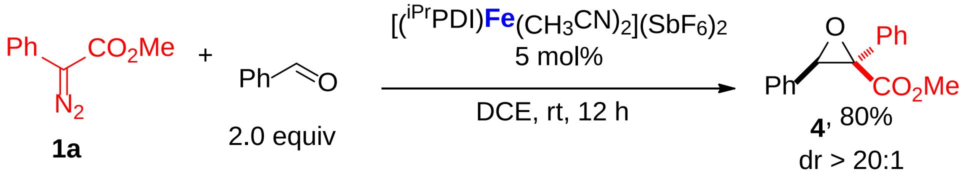

To a two-dram vial containing a magnetic stirring bar, $[(^{iPr}PDI)Fe(CH_3CN)_2](SbF_6)_2$ (21.8 mg, 0.020 mmol, 5 mol%) and 2.0 mL of dry 1,2-dichloroethane (DCE) were added sequentially under a nitrogen atmosphere. Then benzaldehyde (85.0 mg, 0.8 mmol, 2.0 equiv.) was subjected into the system. The flask was capped by a rubber septum, and its solution was stirred before **1a** (70.5 mg, 0.40 mmol, 1.0 equiv.) dissolved in 1.0 mL of dry DCE was added. Stirring was continued at room temperature until all the diazo compound **1a** was consumed based on TLC analysis. Subsequently, the reaction mixture was passed through a short pad of Celite and concentrated under reduced pressure. The diastereomeric ratio was determined by ^{1}H NMR spectroscopy of the residue, which was then purified by flash chromatography on silica gel using 10:1 hexanes:ethyl acetate as the eluent to afford the epoxide product **4** (80% yield, 81.4 mg). Analytical data for **4** is listed below.

(±) (2R,3R)-methyl 2,3-diphenyloxirane-2-carboxylate 4.

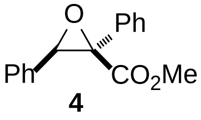

Colorless oil, 80% yield, 81.4 mg, dr > 20:1. ^{1}H NMR (500 MHz, $CDCl_3$) δ 7.66 – 7.62 (comp, 2H), 7.42 – 7.34 (comp, 8H), 4.15 (s, 1H), 3.55 (s, 3H). ^{13}C NMR (126 MHz, $CDCl_3$) δ 167.18, 134.73, 133.79, 128.93, 128.73, 128.68, 128.47, 126.28, 126.04, 67.14, 65.91, 52.32. HRMS (ESI) calculated for $C_{16}H_{14}O_3Cs$ $[M+Cs]^+$: 386.9997; found: 387.0001. The spectroscopic date is consistent with previously reported results.[11]

Procedures for the Bis(arylimino)pyridine Iron catalyzed Doyle–Kirmse Reaction of 1a and Allyl Phenyl Sulfide.

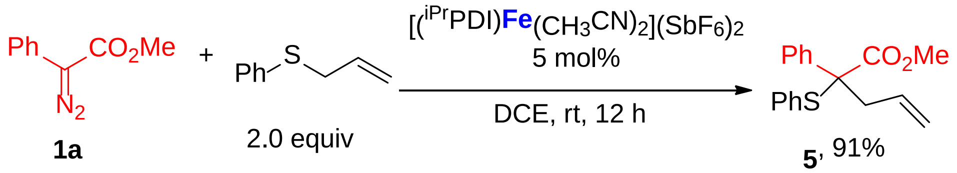

To a two-dram vial containing a magnetic stirring bar, $[(^{iPr}PDI)Fe(CH_3CN)_2](SbF_6)_2$ (21.8 mg, 0.020 mmol, 5 mol%) and 2.0 mL of dry 1,2-dichloroethane (DCE) were added sequentially under a nitrogen atmosphere. Then allyl phenyl sulfide (120.2 mg, 0.8 mmol, 2.0 equiv.) was subjected into the system. The flask was capped by a rubber septum, and its solution was stirred before **1a** (70.5 mg, 0.40 mmol, 1.0 equiv.) dissolved in 1.0 mL of dry DCE was added. Stirring was continued at room temperature until all the diazo compound **1a** was consumed based on TLC analysis. Subsequently, the reaction mixture was passed through a short pad of Celite and concentrated under reduced pressure. The diastereomeric ratio was determined by ^{1}H NMR spectroscopy of the residue, which was then purified by flash chromatography on silica gel using 10:1 hexanes:ethyl acetate as the eluent to afford the epoxide product **5** (91% yield, 108.1 mg). Analytical data for **5** is listed below.

Methyl 2-phenyl-2-(phenylthio)pent-4-enoate 5.

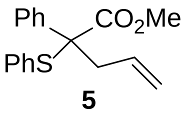

5

Colorless oil, 91% yield, 108.1 mg. ^{1}H NMR (500 MHz, $CDCl_3$) δ 7.31 – 7.24 (comp, 7H), 7.20 – 7.16 (comp, 3H), 5.99 – 5.85 (m, 1H), 5.11 (ddt, J = 10.2, 2.0, 1.1 Hz, 1H), 5.06 (ddd, J = 17.1, 3.3, 1.5 Hz, 1H), 3.70 (s, 3H), 2.93 – 2.80 (comp, 2H). ^{13}C NMR (126 MHz, $CDCl_3$) δ 172.46, 139.85, 136.99, 133.27, 130.72, 129.39, 128.60, 128.19, 127.62, 127.51, 118.93, 64.60, 52.76, 40.69. HRMS (ESI) calculated for $C_{18}H_{18}O_2SCs$ $[M+Cs]^+$: 431.0082; found: 431.0086. The spectroscopic date is consistent with previously reported results.[12]

Procedures for the Bis(arylimino)pyridine Iron catalyzed N–H insertion of 1a and Aniline.

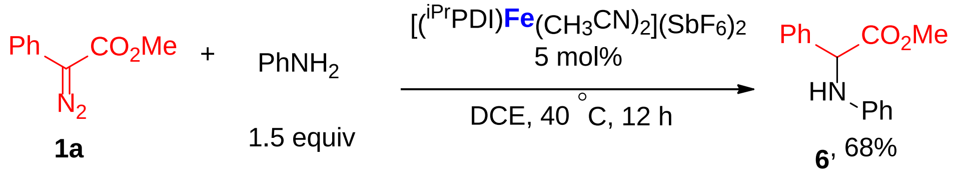

To a two-dram vial containing a magnetic stirring bar, $[(^{iPr}PDI)Fe(CH_3CN)_2](SbF_6)_2$ (21.8 mg, 0.020 mmol, 5 mol%) and 2.0 mL of dry 1,2-dichloroethane (DCE) were added sequentially under a nitrogen atmosphere. Then aniline (56.0 mg, 0.6 mmol, 1.5 equiv.) was subjected into the system. The flask was capped by a rubber septum, and its solution was stirred before **1a** (70.5 mg, 0.40 mmol, 1.0 equiv.) dissolved in 1.0 mL of dry DCE was added. Stirring was continued at 40 °C until all the diazo compound **1a** was consumed based on TLC analysis. Subsequently, the reaction mixture was passed through a short pad of Celite and concentrated under reduced pressure. The residue was then purified by flash chromatography on silica gel using 10:1 hexanes:ethyl acetate as the eluent to afford the N–H insertion product **6** (68% yield, 65.7 mg). Analytical data for **6** is listed below.

Methyl 2-phenyl-2-(phenylamino)acetate 6.

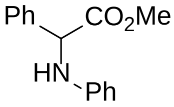

6

White solid, 68% yield, 65.7 mg. ^{1}H NMR (500 MHz, $CDCl_3$) δ 7.52 – 7.48 (comp, 2H), 7.37 – 7.28 (comp, 3H), 7.12 (dd, J = 8.4, 7.4 Hz, 2H), 6.71 – 6.68 (m, 1H), 6.59 – 6.52 (comp, 2H), 5.07 (d, J = 5.8 Hz, 1H), 4.95 (d, J = 5.3 Hz, 1H), 3.73 (s, 3H). ^{13}C NMR (126 MHz, $CDCl_3$) δ 172.42, 145.99, 137.67, 129.34, 128.98, 128.41, 127.34, 118.19, 113.46, 60.79, 52.94. HRMS (ESI) calculated for $C_{15}H_{16}NO_2$ $[M+H]^+$: 242.1181; found: 242.1186. The spectroscopic date is consistent with previously reported results.[13]

Procedures for the Bis(arylimino)pyridine Iron catalyzed C–H insertion of 1a and *N,N*-Dimethylaniline.

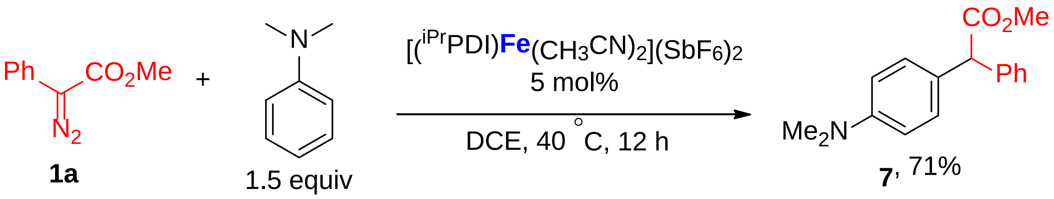

To a two-dram vial containing a magnetic stirring bar, $[(^{iPr}PDI)Fe(CH_3CN)_2](SbF_6)_2$ (21.8 mg,

0.020 mmol, 5 mol%) and 2.0 mL of dry 1,2-dichloroethane (DCE) were added sequentially under a nitrogen atmosphere. Then *N*,*N*-dimethylaniline (72.7 mg, 0.6 mmol, 1.5 equiv.) was subjected into the system. The flask was capped by a rubber septum, and its solution was stirred before **1a** (70.5 mg, 0.40 mmol, 1.0 equiv.) dissolved in 1.0 mL of dry DCE was added. Stirring was continued at 40 °C until all the diazo compound **1a** was consumed based on TLC analysis. Subsequently, the reaction mixture was passed through a short pad of Celite and concentrated under reduced pressure. The residue was then purified by flash chromatography on silica gel using 10:1 hexanes:ethyl acetate as the eluent to afford the N–H insertion product **7** (71% yield, 76.6 mg). Analytical data for **6** is listed below.

Methyl 2-[4-(dimethylamino)phenyl]-2-phenylacetate 7.

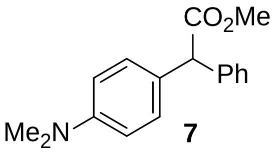

7

White solid, 71% yield, 76.6 mg. ^{1}H NMR (500 MHz, $CDCl_3$) δ 7.31 – 7.30 (comp, 4H), 7.26 – 7.24 (comp, 1H), 7.18 (d, J = 8.8 Hz, 2H), 6.69 (d, J = 8.8 Hz, 2H), 4.95 (s, 1H), 3.73 (s, 3H), 2.92 (s, 6H). ^{13}C NMR (126 MHz, $CDCl_3$) δ 173.65, 149.85, 139.49, 129.35, 128.58, 128.56, 127.10, 126.41, 112.67, 56.22, 52.29, 40.65. HRMS (ESI) calculated for $C_{17}H_{20}NO_2$ $[M+H]^+$: 270.1494; found: 270.1497. The spectroscopic date is consistent with previously reported results.[14]

General Procedures for the Bis(arylimino)pyridine Iron catalyzed O–H insertion of 1a and Alcohols/water.

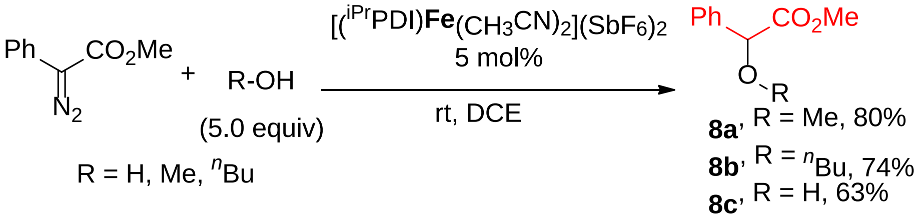

To a two-dram vial containing a magnetic stirring bar, $[(^{iPr}PDI)Fe(CH_3CN)_2](SbF_6)_2$ (21.8 mg, 0.020 mmol, 5 mol%) and 2.0 mL of dry 1,2-dichloroethane (DCE) were added sequentially under a nitrogen atmosphere. Then alcohol (2.0 mmol, 5.0 equiv.) was subjected into the system. The flask was capped by a rubber septum, and its solution was stirred before **1a** (70.5 mg, 0.40 mmol, 1.0 equiv.) dissolved in 1.0 mL of dry DCE was added. Stirring was continued at room temperature until all the diazo compound **1a** was consumed based on TLC analysis. Subsequently, the reaction

mixture was passed through a short pad of Celite and concentrated under reduced pressure. The residue was then purified by flash chromatography on silica gel using 10:1 hexanes:ethyl acetate as the eluent to afford the O–H insertion product **8.** Analytical data for **8** is listed below.

Methyl 2-methoxy-2-phenylacetate 8a.

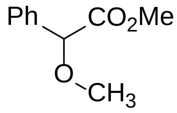

8a

Colorless oil, 80% yield, 57.8 mg. ^{1}H NMR (500 MHz, $CDCl_3$) δ 7.46 – 7.40 (comp, 2H), 7.40 – 7.31 (comp, 3H), 4.77 (s, 1H), 3.71 (s, 3H), 3.40 (s, 3H).^{13}C NMR (126 MHz, $CDCl_3$) δ 171.25, 136.20, 128.91, 128.78, 127.32, 82.63, 57.46, 52.45. HRMS (ESI) calculated for $C_{10}H_{13}O_3$ $[M+H]^+$: 181.0865; found: 181.0868. The spectroscopic date is consistent with previously reported results.[15]

Methyl 2-butoxy-2-phenylacetate 8b.

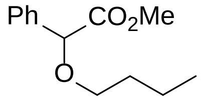

8b

Colorless oil, 80% yield, 57.8 mg. ^{1}H NMR (500 MHz, $CDCl_3$) δ 7.46 – 7.42 (comp, 2H), 7.38 –7.30 (comp, 3H), 4.87 (s, 1H), 3.68 (s, 3H), 3.58 –3.52 (m, 1H), 3.49 –3.41 (m, 1H), 1.68 –1.64 (comp, 2H), 1.42 –1.37 (comp, 2H) 0.91 (t, J = 7.5 Hz, 3H). ^{13}C NMR (126 MHz, $CDCl_3$) δ 171.68, 136.92, 128.73, 128.61, 127.24, 81.39, 70.00, 52.28, 31.81, 19.29, 13.94. HRMS (ESI) calculated for $C_{13}H_{19}O_3$ $[M+H]^+$: 233.1334; found: 2339.1335. The spectroscopic date is consistent with previously reported results.[15]

Methyl 2-hydroxy-2-phenylacetate 8c.

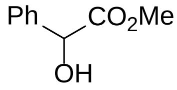

8c

Colorless oil, 63% yield, 42.0 mg. ^{1}H NMR (500 MHz, $CDCl_3$) δ 7.46–7.35 (comp, 5H), 5.43 (s, 1H), 3.76 (s, 3H), 3.73 (d, J = 4.8 Hz, 1H). The spectroscopic date is consistent with previously reported results. [15]

Mechanistic Study

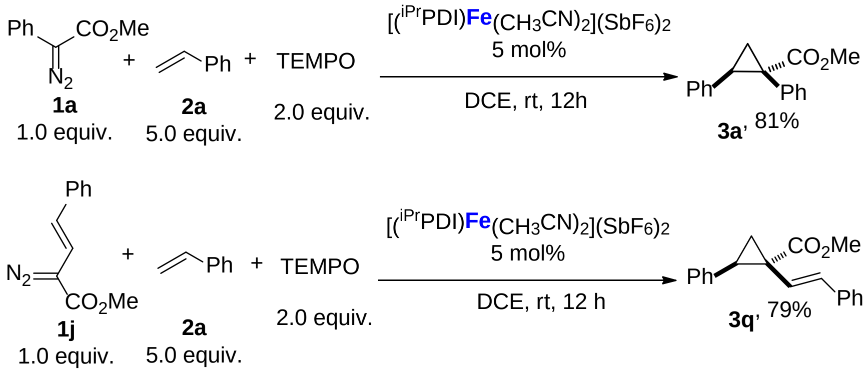

To a two-dram vial containing a magnetic stirring bar, $[(^{iPr}PDI)Fe(CH_3CN)_2](SbF_6)_2$ (21.8 mg, 0.020 mmol, 5 mol%) and 2.0 mL of dry 1,2-dichloroethane (DCE) were added sequentially under a nitrogen atmosphere. Then styrene **2a** (208.3 mg, 2.0 mmol, 5.0 equiv.) and 2,2,6,6-tetramethylpiperidine *N*-oxide (125.0 mg, TEMPO, 0.8 mmol, 2.0 equiv.) was subjected into the system. The flask was capped by a rubber septum, and its solution was stirred before diazo compound **1a** (70.5 mg, 0.40 mmol, 1.0 equiv.) dissolved in 1.0 mL of dry DCE was added. Stirring was continued at room temperature for 12 hours. Subsequently, the reaction mixture was passed through a short pad of Celite and concentrated under reduced pressure. The diastereomeric ratio was determined by ^{1}H NMR spectroscopy of the residue, which was then purified by flash chromatography on silica gel using 10:1 hexanes:ethyl acetate as the eluent to afford the corresponding cyclopropanation product **3**.

References:

1. (a) Davies, H. M. L.; Hansen, T.; Churchill, M. R. *J. Am. Chem. Soc.* **2000**, *122*, 3063. (b) Doyle, M. P.; Yan, M.; Hu, W.; Gronenberg, L. S. *J. Am. Chem. Soc.* **2003**, *125*, 4692.
2. (a) Aranda, C.; Cornejo, A.; Fraile, J. M.; García-Verdugo, E.; Gil, M. J.; Luis, S. V.; Mayoral, J. A.; Martinez-Merino, V.; Ochoa, Z. *Green Chemistry* **2011**, *13*, 983. (b) Chen, J.; Xi, T.; Lu, Z. *Organic Chemistry Frontiers* **2018**, *5*, 247.
3. (a) Small, B. L.; Brookhart, M.; Bennett, A. M. A. *J. Am. Chem. Soc.* **1998**, *120*, 4049. (b) Britovsek, G. J. P.; Bruce, M.; Gibson, V. C.; Kimberley, B. S.; Maddox, P. J.; Mastroianni, S.; McTavish, S. J.; Redshaw, C.; Solan, G. A.; Strömberg, S.; White, A. J. P.; Williams, D. J. *J. Am. Chem. Soc.* **1999**, *121*, 8728.
4. (a) Schmidt, R.; Welch, M. B.; Knudsen, R. D.; Gottfried, S.; Alt, H. G. *J. Mol. Catal. A: Chem.* **2004**, *222*, 9. (b) Hoyt, J. M.; Schmidt, V. A.; Tondreau, A. M.; Chirik, P. J. *Science* **2015**, *349*, 960
5. Britovsek, G. J. P.; England, J.; Spitzmesser, S. K.; White, A. J. P.; Williams, D. J. *Dalton Trans.* **2005**, 945.
6. Thompson, J. L.; Davies, H. M. L. *J. Am. Chem. Soc.* **2007**, *129*, 6090.
7. Davies, H. M. L.; Venkataramani, C. *Org. Lett.* **2003**, *5*, 1403.
8. Chepiga, K. M.; Qin, C.; Alford, J. S.; Chennamadhavuni, S.; Gregg, T. M.; Olson, J. P.;

Davies, H. M. L. *Tetrahedron* **2013**, *69*, 5765.

9. (a) Davies, H. M. L.; Panaro, S. A. *Tetrahedron* **2000**, *56*, 4871. (b) Hansen, J.; Li, B.; Dikarev, E.; Autschbach, J.; Davies, H. M. L. *J. Org. Chem.* **2009**, *74*, 6564.
10. L.-A. Liao, F. Zhang, N. Yan, J. A. Golen, J. M. Fox, *Tetrahedron*, 2004, *60*, 1803-1816.
11. Tindall, D. J.; Werlé, C.; Goddard, R.; Philipps, P.; Farès, C.; Fürstner, A. *J. Am. Chem. Soc.* **2018**, *140*, 1884.
12. Hommelsheim, R.; Guo, Y.; Yang, Z.; Empel, C.; Koenigs, R. M. *Angew. Chem. Int. Ed.* **2019**, *58*, 1203.
13. Bartrum, H. E.; Blakemore, D. C.; Moody, C. J.; Hayes, C. J. *Chem. Eur. J.* **2011**, *17*, 9586.
14. Yang, J.-M.; Cai, Y.; Zhu, S.-F.; Zhou, Q.-L. *Org. Biomol. Chem.* **2016**, *14*, 5516.
15. Zhu, S.-F.; Cai, Y.; Mao, H.-X.; Xie, J.-H.; Zhou, Q.-L. *Nat. Chem.* **2010**, *2*, 546.

(*S*)-^{VME}PDI.

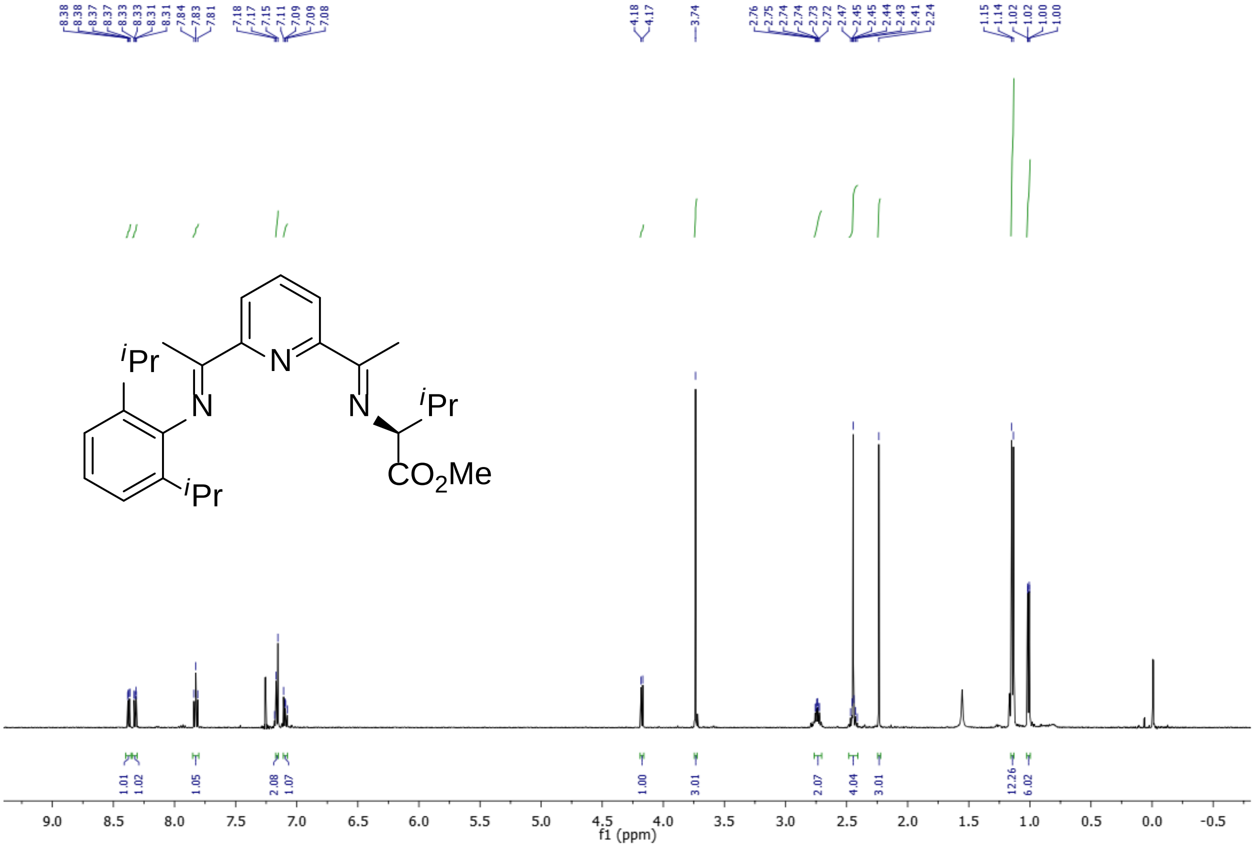

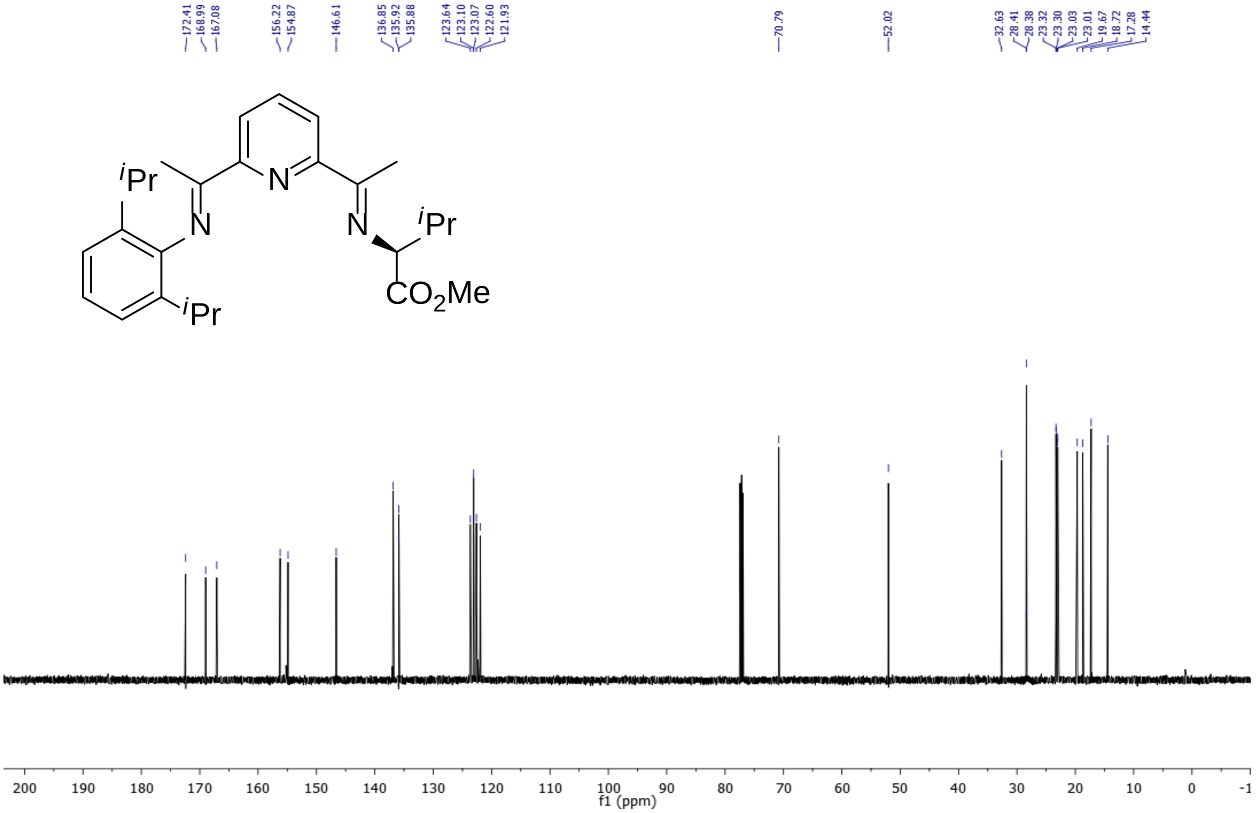

(±) (1S,2R)-Methyl 1,2-diphenylcyclopropanecarboxylate 3a.

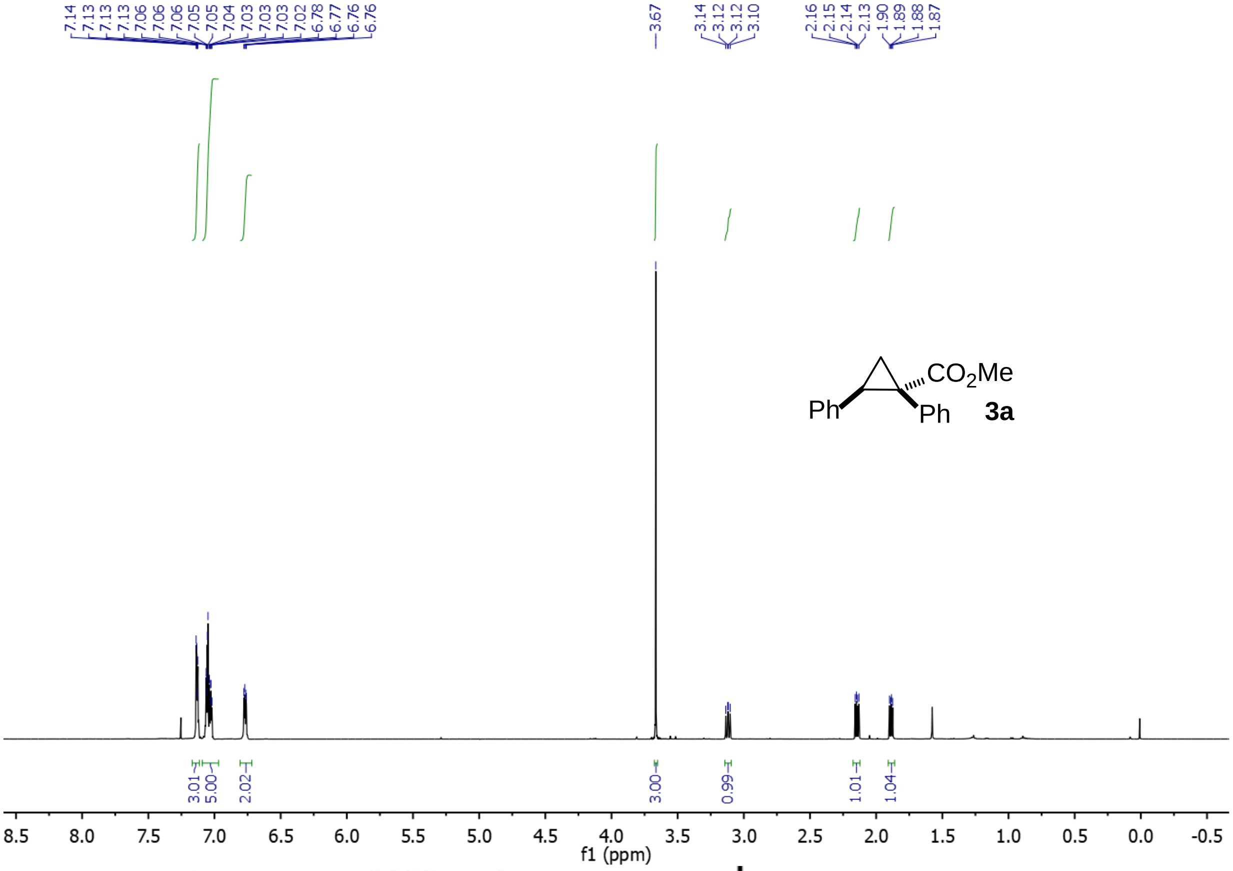

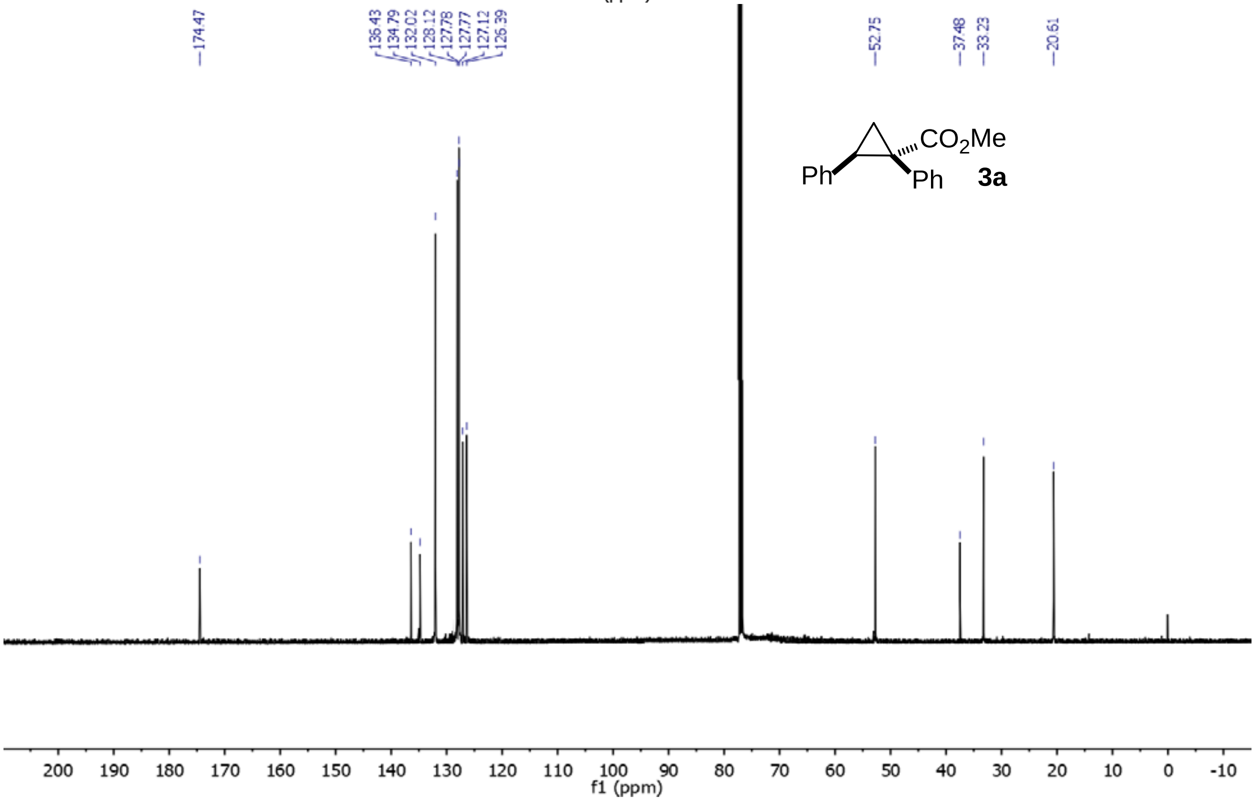

Methyl 7-phenylbicyclo[4.1.0]hept-2-ene-7-carboxylate 3p.

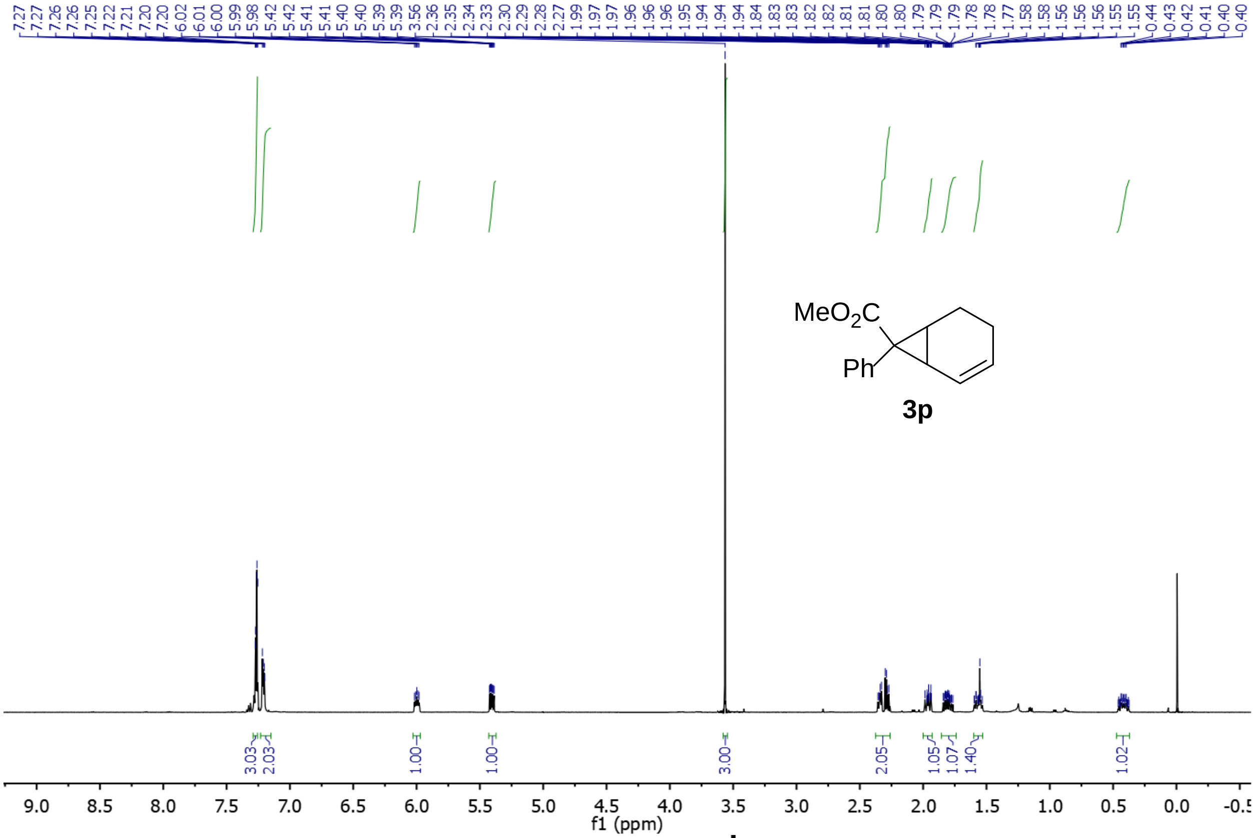

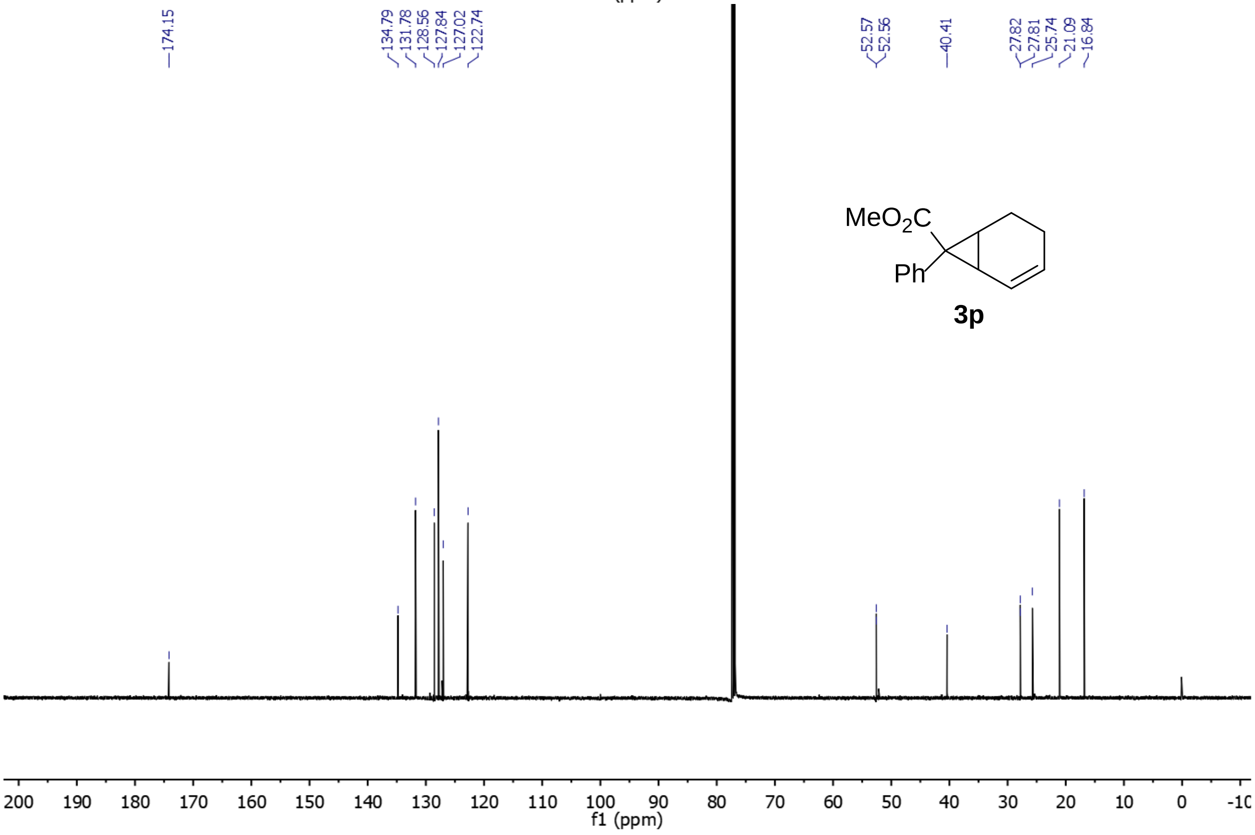

(±) (1R,2R)-methyl 2-phenyl-1-((E)-styryl)cyclopropanecarboxylate 3r.

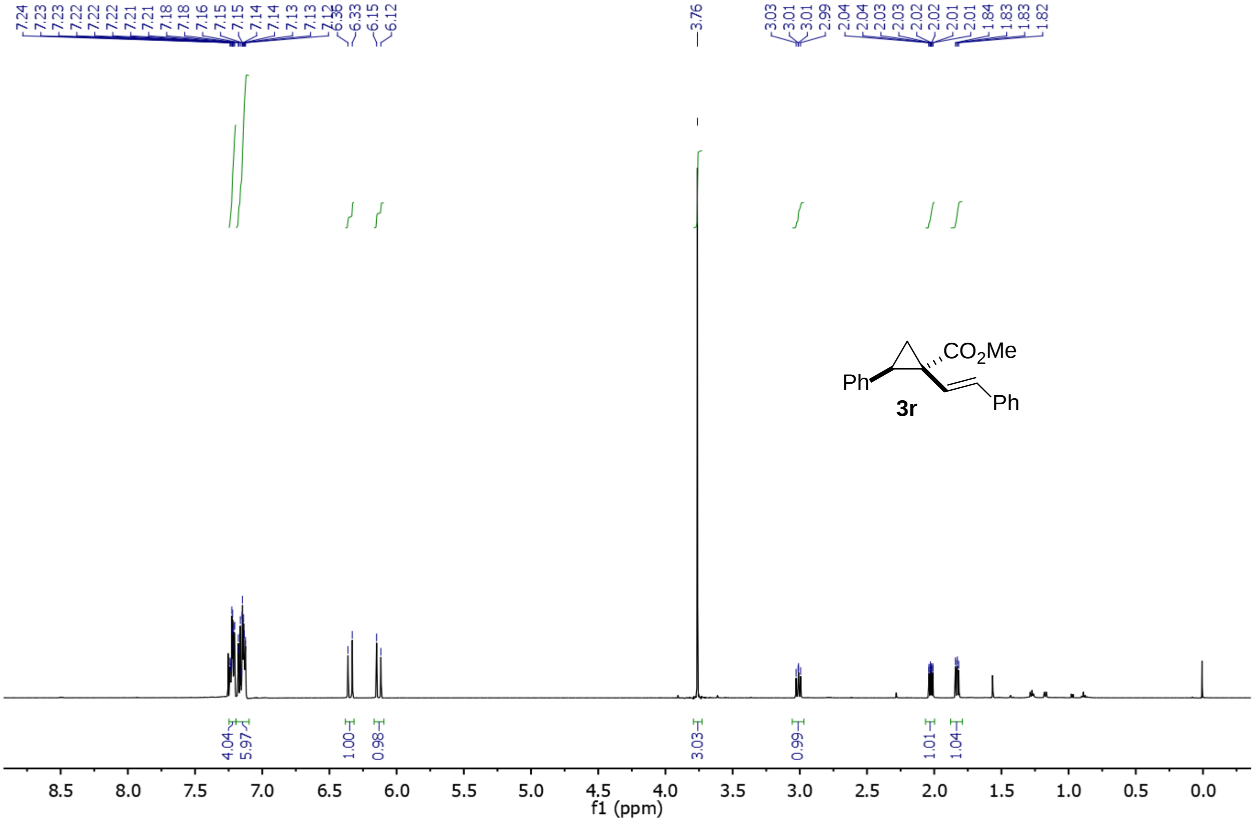

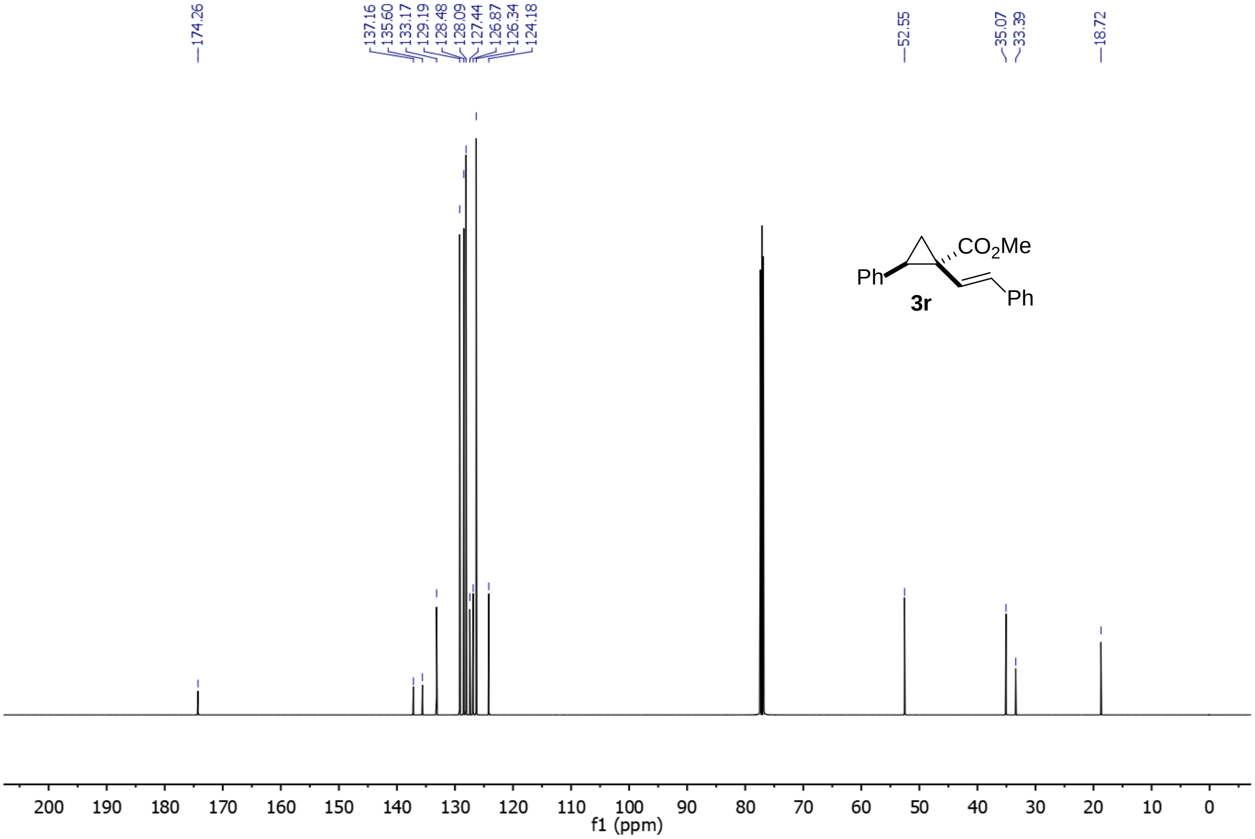

(±) (2R,3R)-methyl 2,3-diphenyloxirane-2-carboxylate 4.

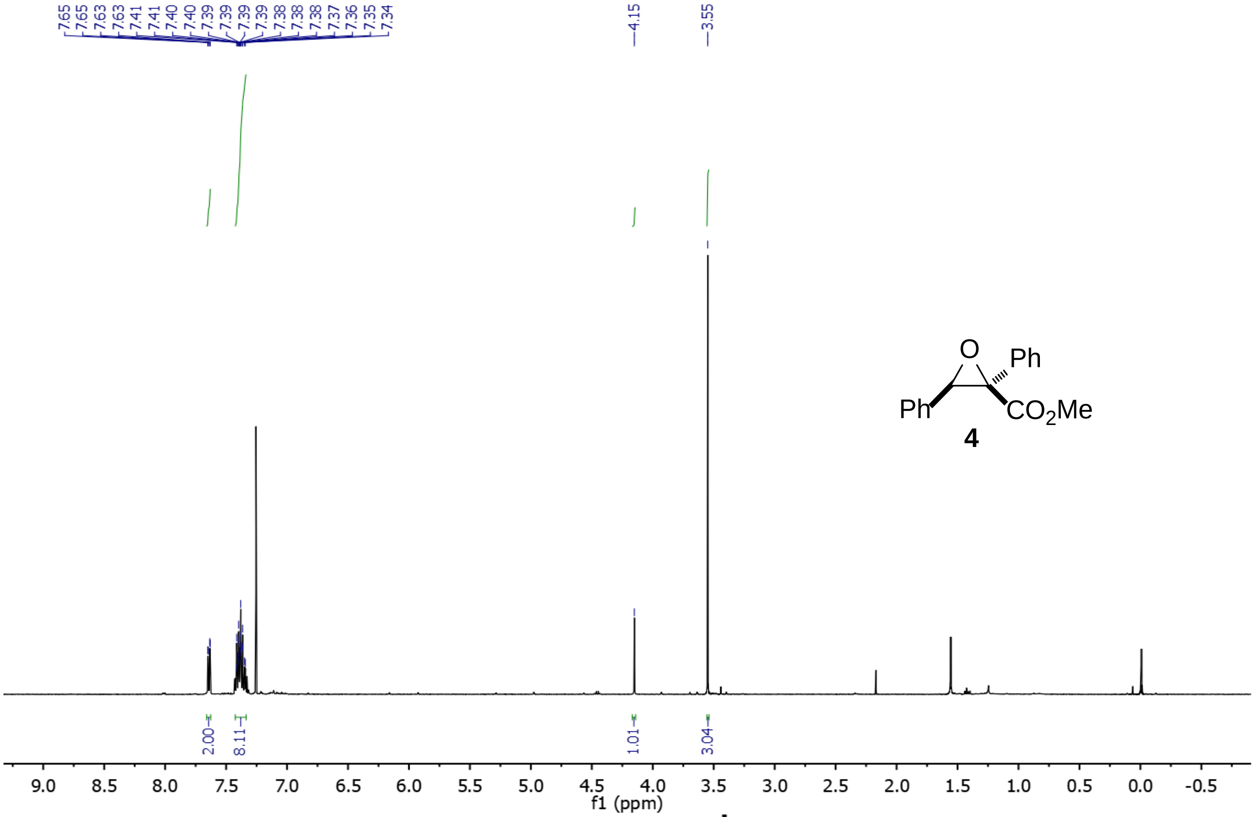

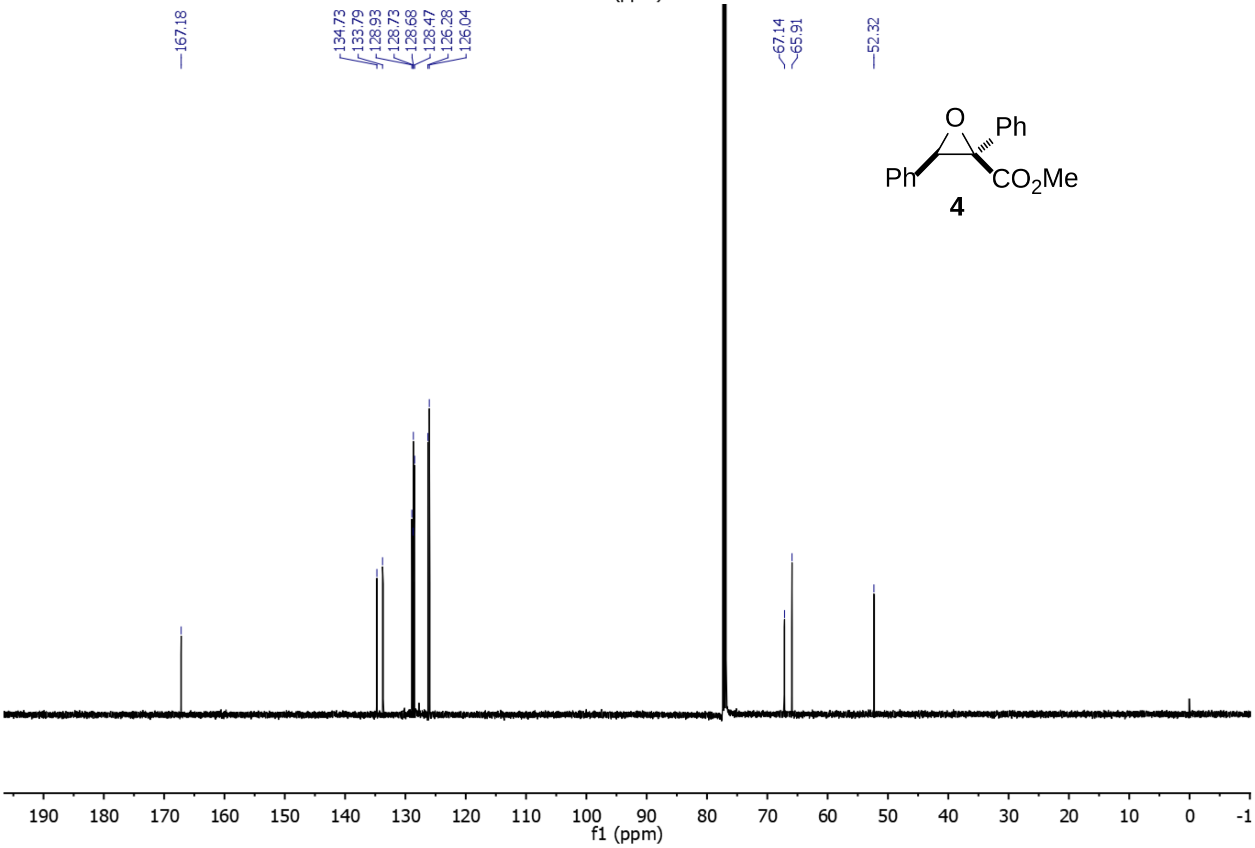

Methyl 2-phenyl-2-(phenylthio)pent-4-enoate 5.

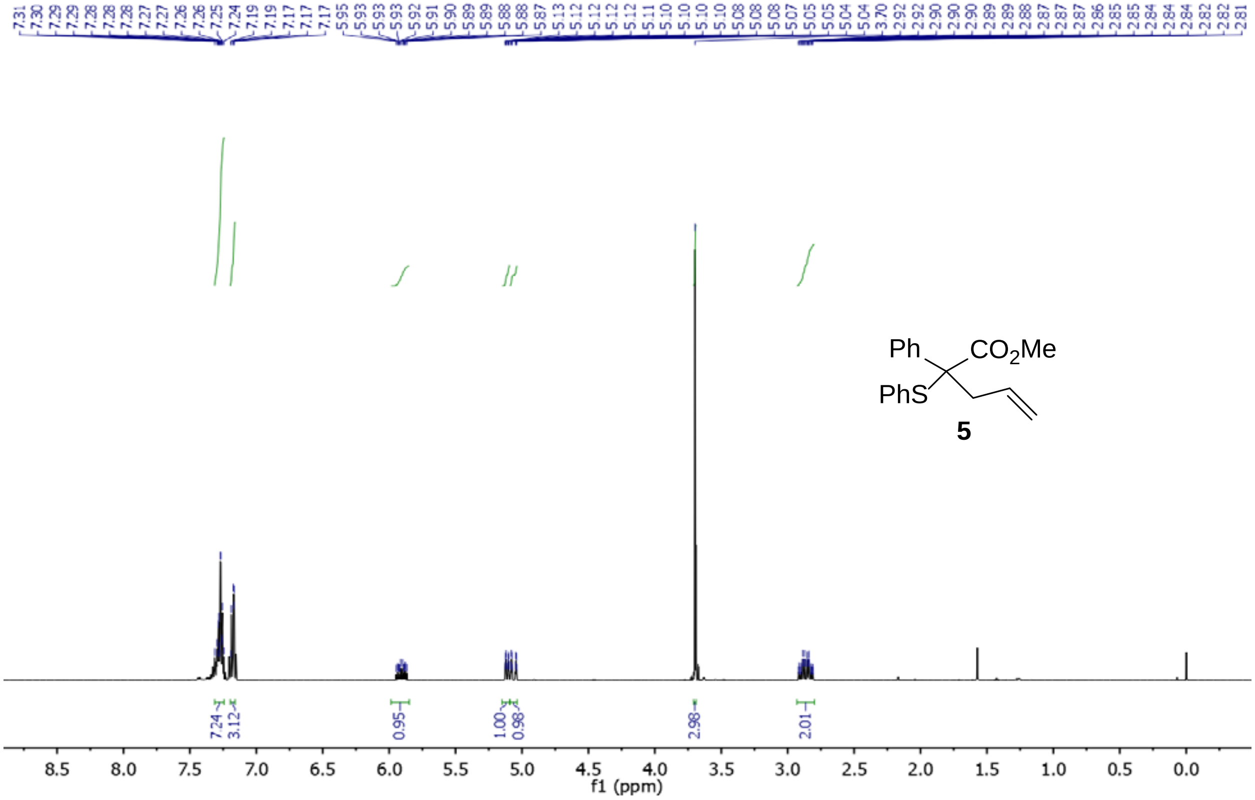

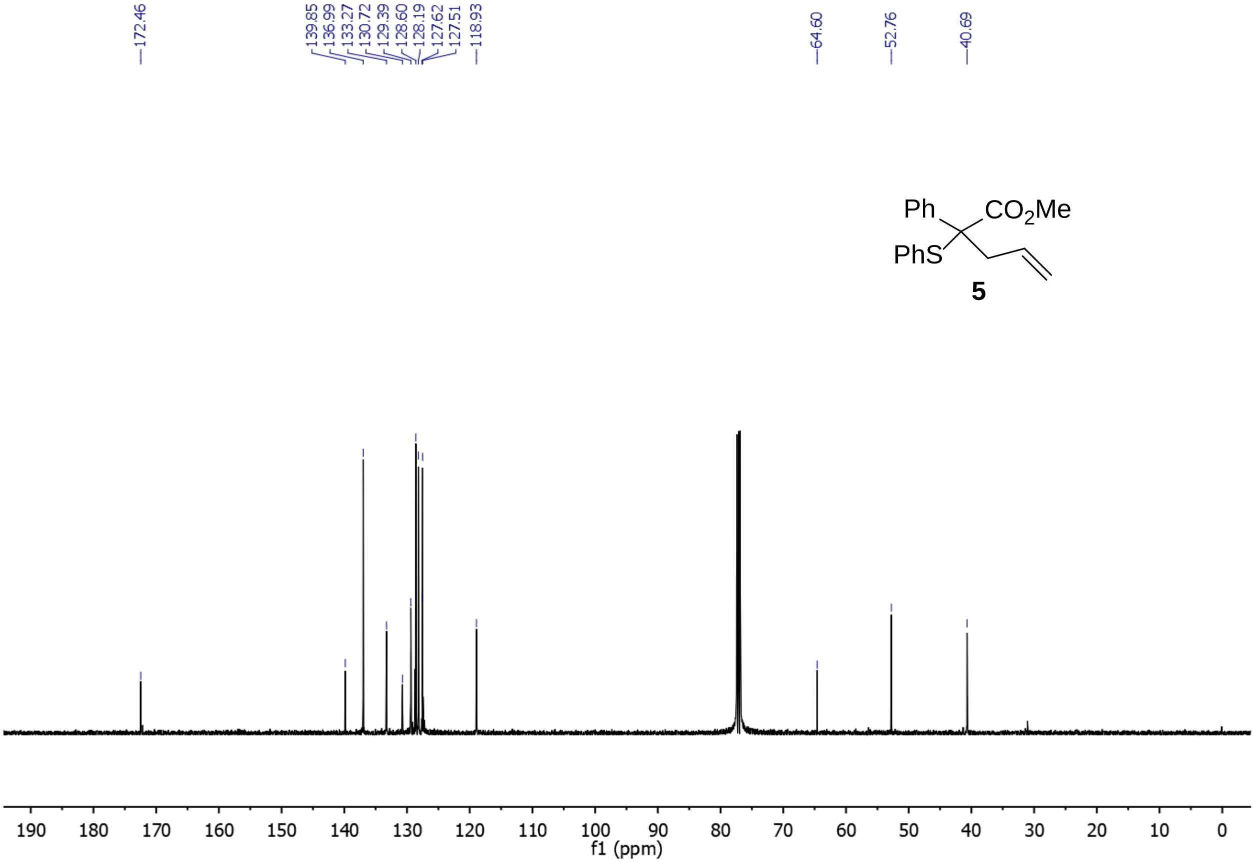

Methyl 2-phenyl-2-(phenylamino)acetate 6.

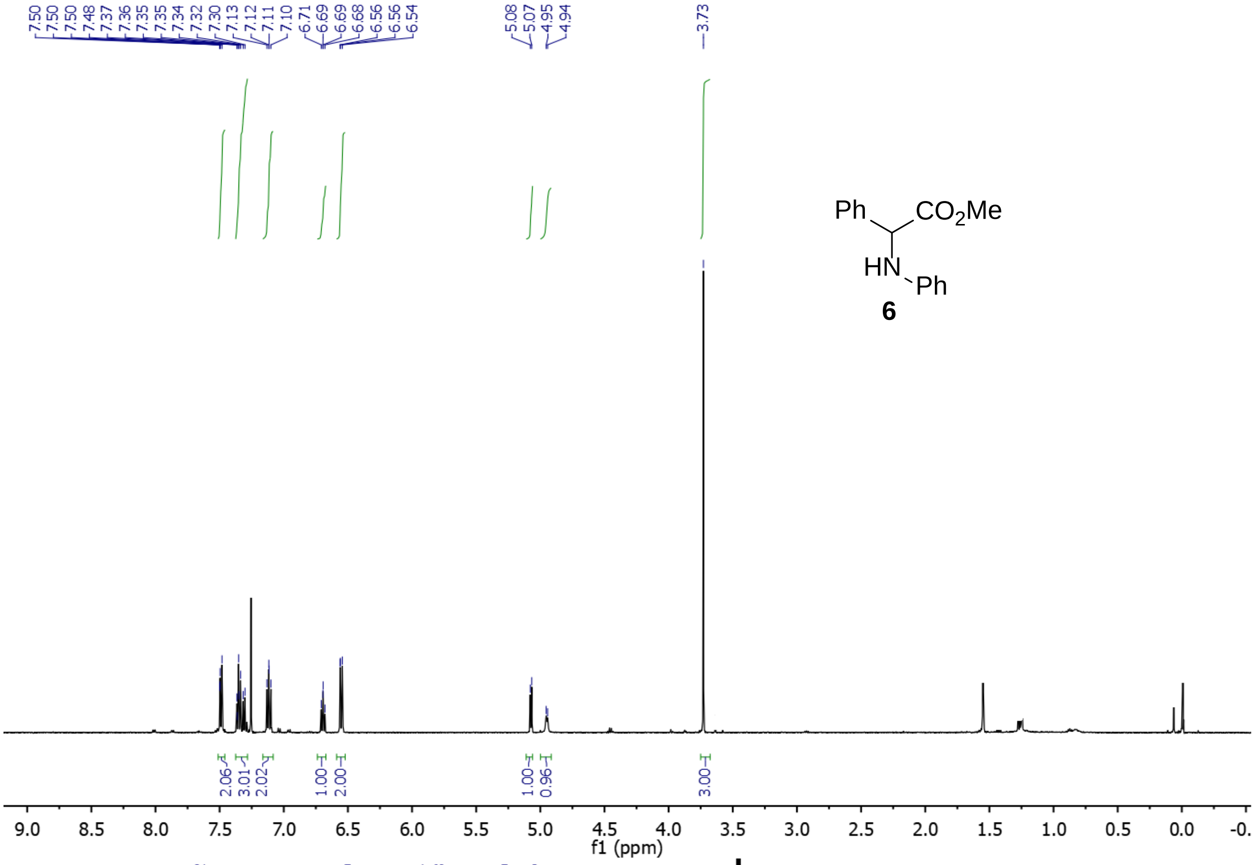

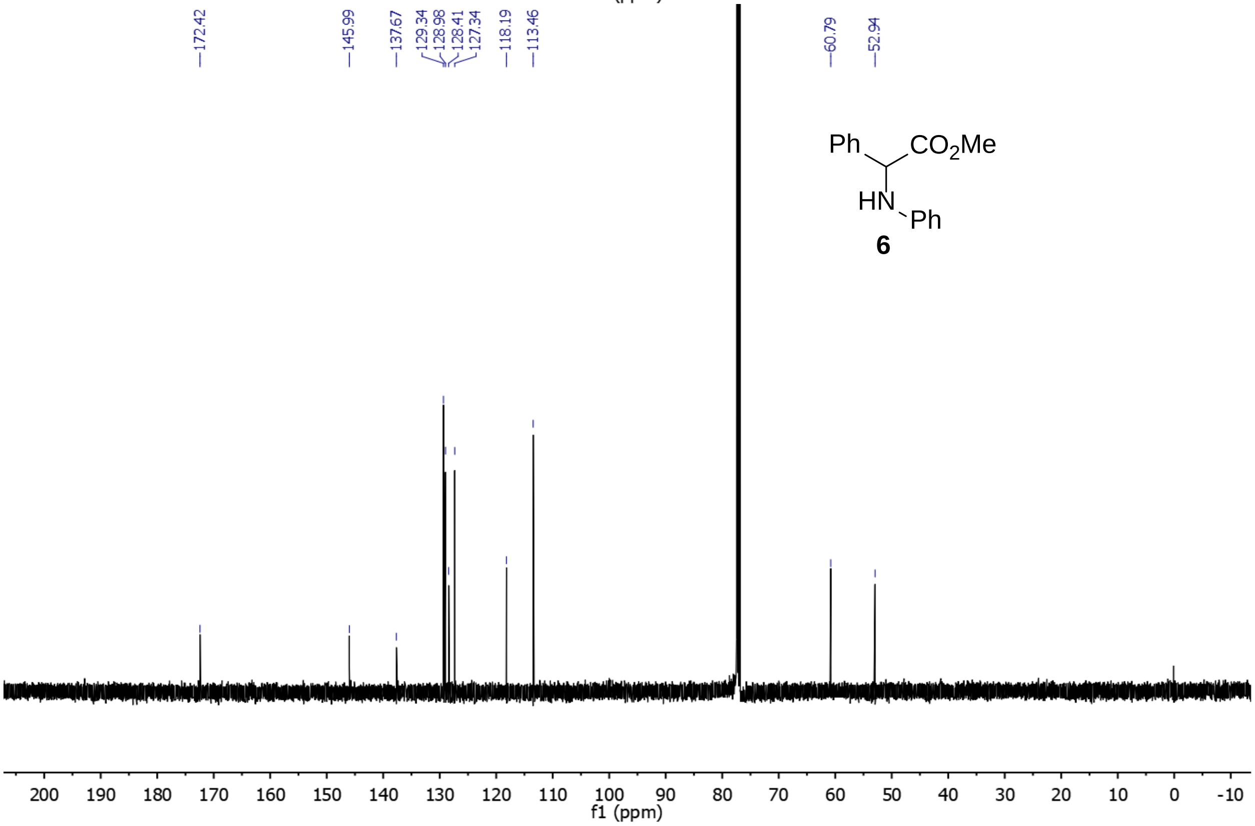

Methyl 2-[4-(dimethylamino)phenyl]-2-phenylacetate 7.

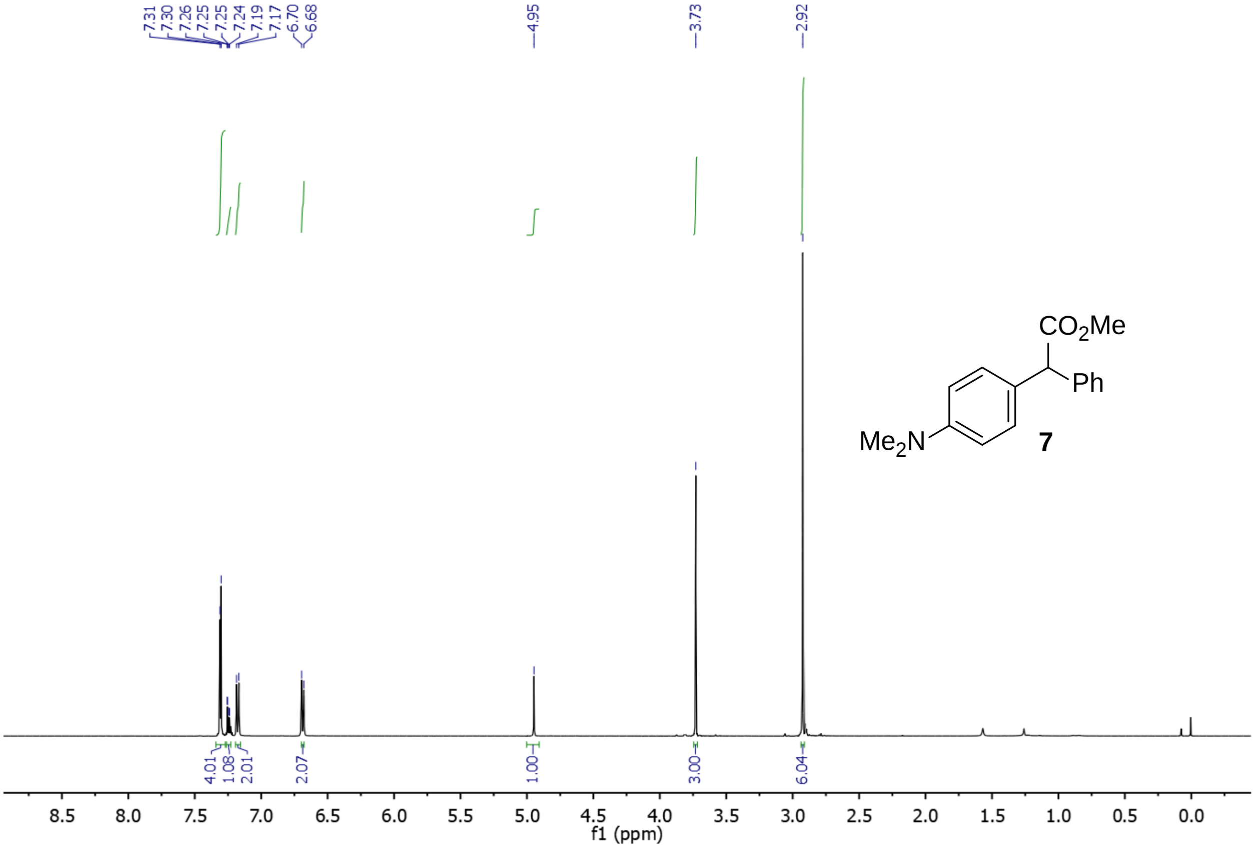

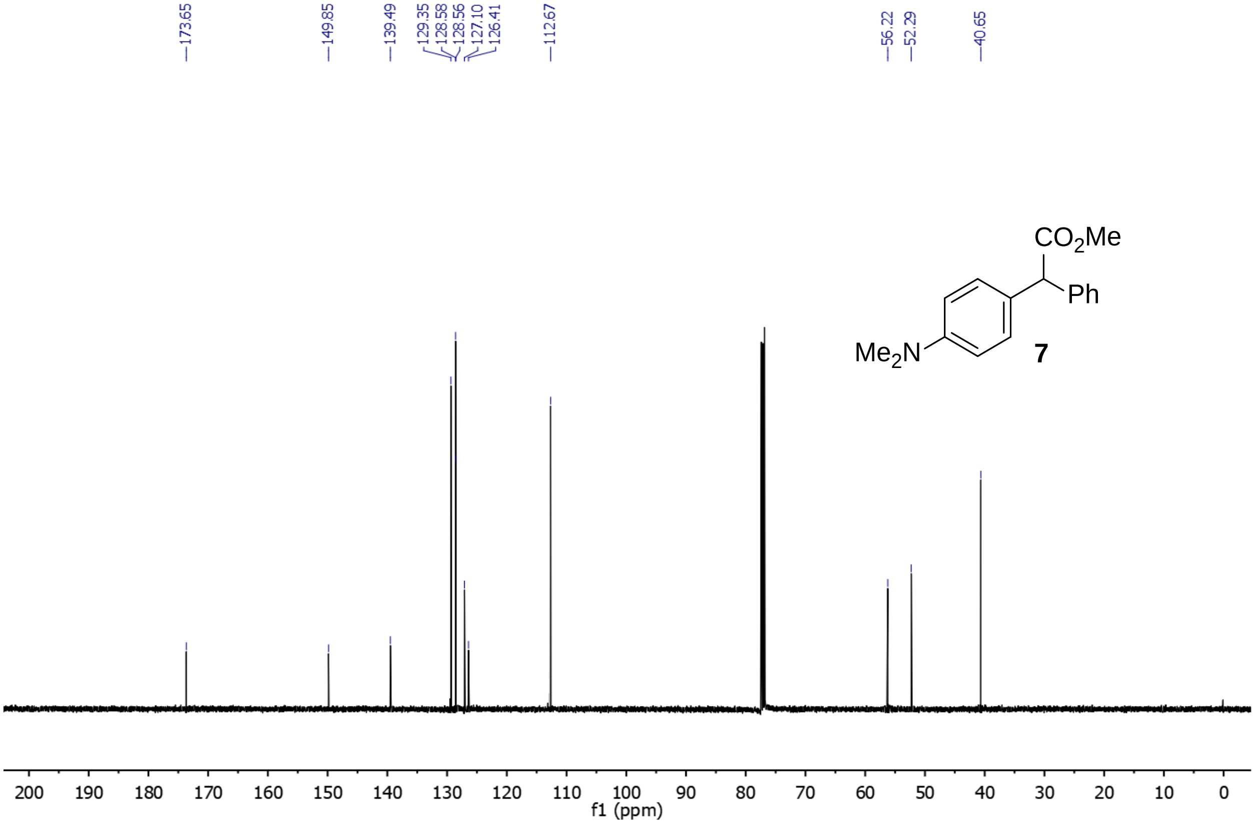

Methyl 2-methoxy-2-phenylacetate 8a.

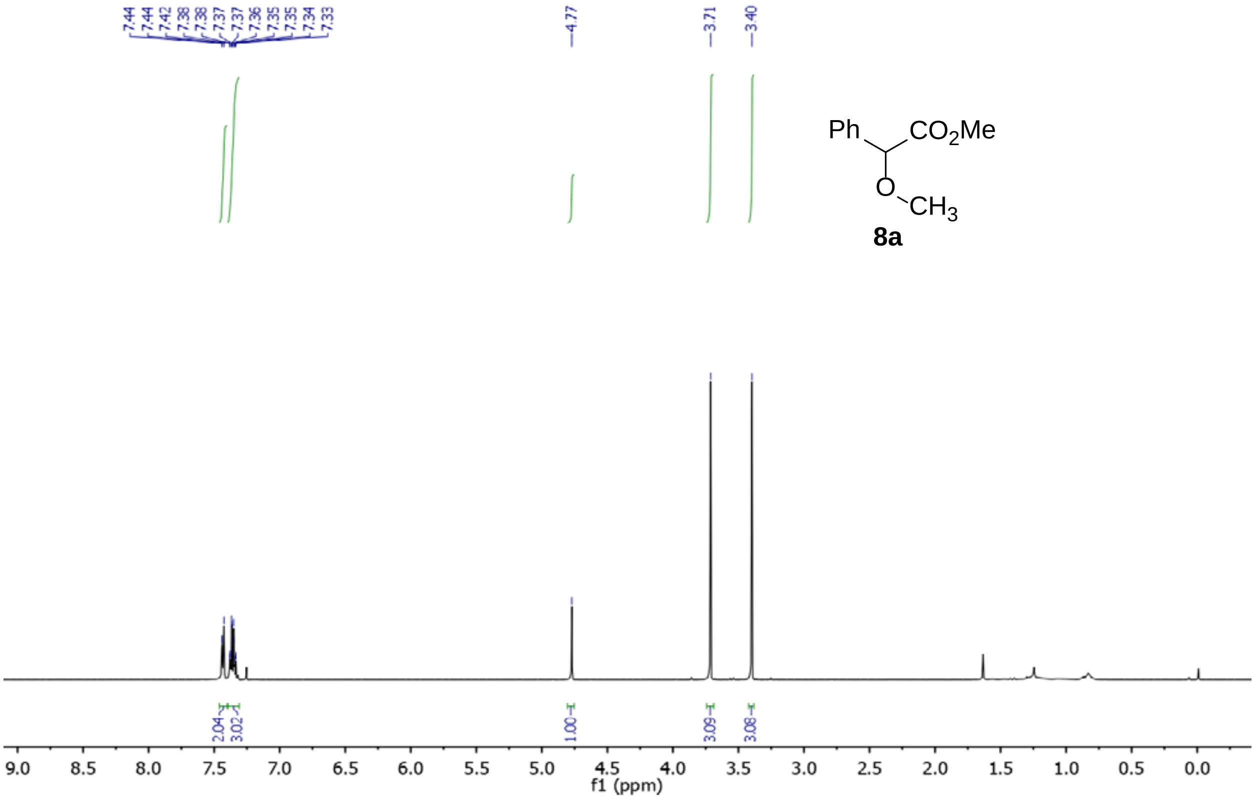

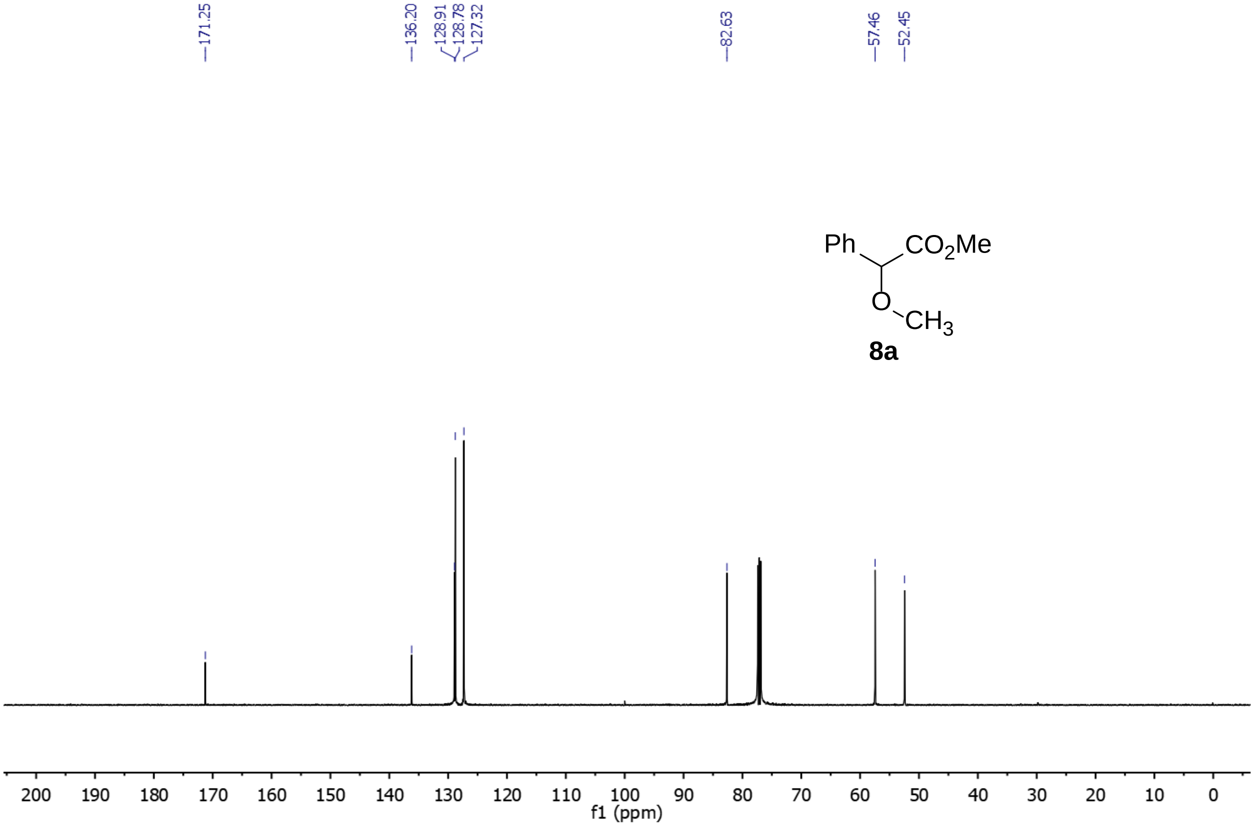

HPLC spectra for (1S,2R)-Methyl 1,2-diphenylcyclopropanecarboxylate 3a. ee = 65%

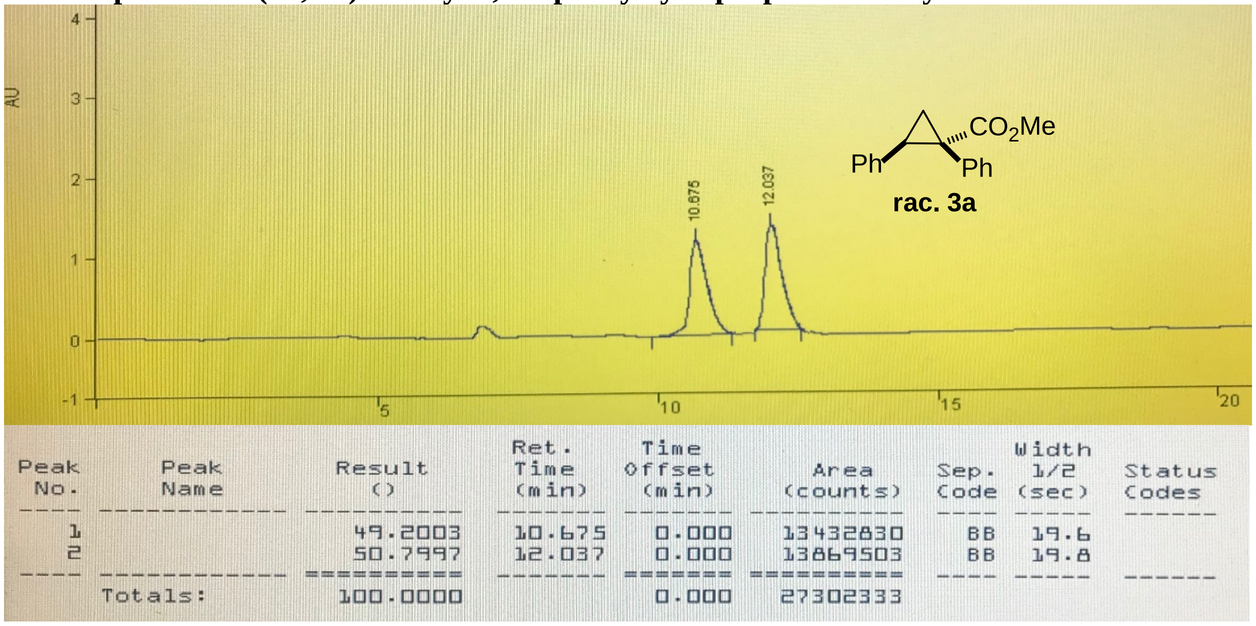

Peak No.	Peak Name	Result ()	Ret. Time (min)	Time Offset (min)	Area (counts)	Sep. Code	Width 1/2 (sec)	Status Codes
1		49.2003	10.675	0.000	13432830	BB	19.6	
2		50.7997	12.037	0.000	13869503	BB	19.8	
	Totals:	100.0000		0.000	27302333			

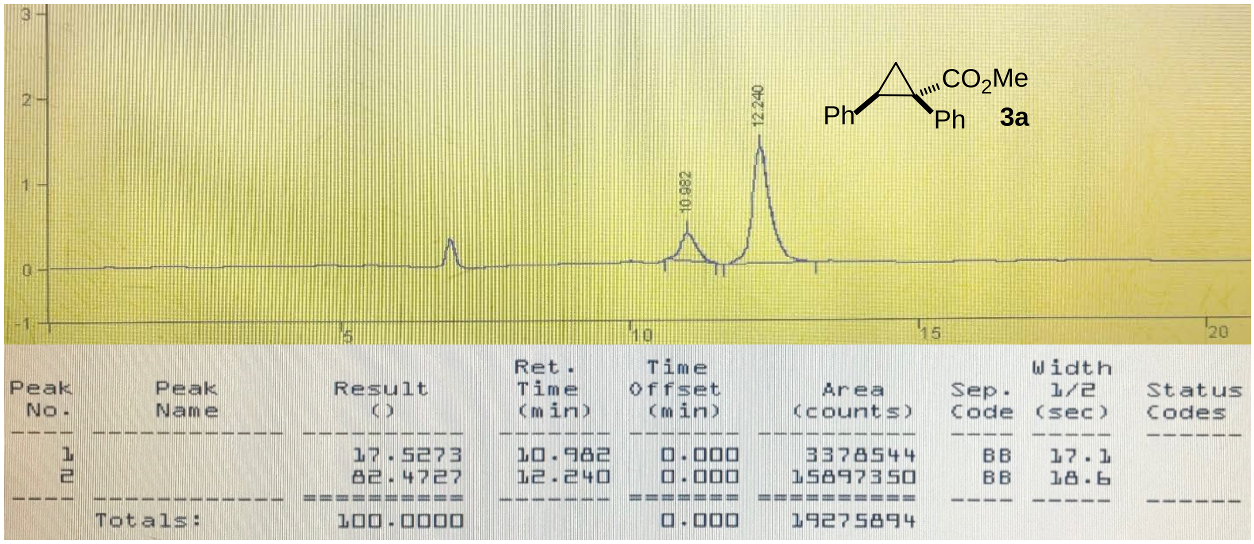

Peak No.	Peak Name	Result ()	Ret. Time (min)	Time Offset (min)	Area (counts)	Sep. Code	Width 1/2 (sec)	Status Codes
1		17.5273	10.982	0.000	3378544	BB	17.1	
2		82.4727	12.240	0.000	15897350	BB	18.6	
	Totals:	100.0000		0.000	19275894			
